# Supplementary material for: Profound phenotypic and epigenetic heterogeneity of the HIV-1-infected CD4+ T cell reservoir
Source: Nat Immunol. 2022 Dec 19;24(2):359–70. doi: 10.1038/s41590-022-01371-3 (PMC9892009; doi:10.1038/s41590-022-01371-3)
Supplement: Supplementary file 1 — Supplementary Tables 1–9. [file 41590_2022_1371_MOESM1_ESM.pdf]

nature immunology

Resource

https://doi.org/10.1038/s41590-022-01371-3

Profound phenotypic and epigenetic heterogeneity of the HIV-1-infected CD4$^+$ T cell reservoir

In the format provided by the authors and unedited

Supplemental Table 1: Catalog of TotalSeqA antibodies used for study.

DNA_ID	Description	Clone	Barcode
A0006	CD86	IT2.2	GTCTTTGTCAGTGCA
A0007	CD274 (B7-H1, PD-L1)	29E.2A3	GTTGTCCGACAATAC
A0020	CD270 (HVEM, TR2)	122	TGATAGAAACAGACC
A0023	CD155 (PVR)	SKII.4	ATCACATCGTTGCCA
A0024	CD112 (Nectin-2)	TX31	AACCTTCCGTCTAAG
A0026	CD47	CC2C6	GCATTCTGTCACCTA
A0029	CD48	BJ40	CTACGACGTAGAAGA
A0031	CD40	5C3	CTCAGATGGAGTATG
A0032	CD154	24-31	GCTAGATAGATGCAA
A0033	CD52	HI186	CTTTGTACGAGCAAA
A0034	CD3	UCHT1	CTCATTGTAACTCCT
A0046	CD8	SK1	GCGCAACTTGATGAT
A0047	CD56 (NCAM)	5.1H11	TCCTTTCCTGATAGG
A0050	CD19	HIB19	CTGGGCAATTACTCG
A0052	CD33	P67.6	TAACTCAGGGCCTAT
A0053	CD11c	S-HCL-3	TACGCCTATAACTTG
A0058	HLA-A,B,C	W6/32	TATGCGAGGCTTATC
A0063	CD45RA	HI100	TCAATCCTTCCGCTT
A0064	CD123	6H6	CTTCACTCTGTCAGG
A0066	CD7	CD7-6B7	TGGATTCCCGGACTT
A0070	CD49f	GoH3	TTCCGAGGATGATCT
A0071	CD194 (CCR4)	L291H4	AGCTTACCTGCACGA
A0072	CD4	RPA-T4	TGTTCCCGCTCAACT
A0073	CD44	IM7	TGGCTTCAGGTCCTA
A0081	CD14	M5E2	TCTCAGACCTCCGTA
A0083	CD16	3G8	AAGTTCACTCTTTGC
A0085	CD25	BC96	TTTGTCCTGTACGCC
A0087	CD45RO	UCHL1	CTCCGAATCATGTTG
A0088	CD279 (PD-1)	EH12.2H7	ACAGCGCCGTATTTA
A0089	TIGIT (VSTM3)	A15153G	TTGCTTACCGCCAGA
A0090	Mouse IgG1, κ isotype Ctrl	MOPC-21	GCCGGACGACATTAA
A0091	Mouse IgG2a, κ isotype Ctrl	MOPC-173	CTCCTACCTAAACTG
A0092	Mouse IgG2b, κ isotype Ctrl	MPC-11	ATATGTATCACGCGA
A0095	Rat IgG2b, κ Isotype Ctrl	RTK4530	GATTCTTGACGACCT
A0100	CD20	2H7	TTCTGGGTCCCTAGA
A0101	CD335 (NKp46)	9E2	ACAATTTGAACAGCG
A0124	CD31	WM59	ACCTTTATGCCACGG
A0127	Podoplanin	NC-08	GGTTACTCGTTGTGT
A0134	CD146	P1H12	CCTTGGATAACATCA
A0136	IgM	MHM-88	TAGCGAGCCCGTATA
A0138	CD5	UCHT2	CATTAACGGGATGCC
A0140	CD183 (CXCR3)	G025H7	GCGATGGTAGATTAT
A0141	CD195 (CCR5)	J418F1	CCAAAGTAAGAGCCA
A0142	CD32	FUN-2	GCTTCCGAATTACCG
A0143	CD196 (CCR6)	G034E3	GATCCCTTTGTCACT
A0144	CD185 (CXCR5)	J252D4	AATTCAACCGTCGCC
A0145	CD103 (Integrin αE)	Ber-ACT8	GACCTCATTGTGAAT
A0146	CD69	FN50	GTCTCTTGGCTTAAA
A0147	CD62L	DREG-56	GTCCCTGCAACTTGA
A0149	CD161	HP-3G10	GTACGCAGTCCTTCT
A0151	CD152 (CTLA-4)	BNI3	ATGGTTCACGTAATC
A0152	CD223 (LAG-3)	11C3C65	CATTTGTCTGCCGGT

(continued)

DNA_ID	Description	Clone	Barcode
A0153	KLRG1 (MAFA)	SA231A2	CTTATTTCCTGCCCT
A0154	CD27	O323	GCACTCCTGCATGTA
A0155	CD107a (LAMP-1)	H4A3	CAGCCCACTGCAATA
A0156	CD95 (Fas)	DX2	CCAGCTCATTAGAGC
A0158	CD134 (OX40)	Ber-ACT35 (ACT35)	AACCCACCGTTGTTA
A0159	HLA-DR	L243	AATAGCGAGCAAGTA
A0160	CD1c	L161	GAGCTACTTCACTCG
A0161	CD11b	ICRF44	GACAAGTGATCTGCA
A0162	CD64	10.1	AAGTATGCCCTACGA
A0163	CD141 (Thrombomodulin)	M80	GGATAACCGCGCTTT
A0165	CD314 (NKG2D)	1D11	CGTGTTTGTTCCTCA
A0167	CD35	E11	ACTTCCGTCGATCTT
A0168	CD57	QA17A04	AACTCCCTATGGAGG
A0170	CD272 (BTLA)	MIH26	GTTATTGGACTAAGG
A0171	CD278 (ICOS)	C398.4A	CGCGCACCCATTAAA
A0172	CD275 (B7-H2, B7-RP1, ICOSL)	9F.8A4	GTTAGTGTTAGCTTG
A0174	CD58 (LFA-3)	TS2/9	GTTCCTATGGACGAC
A0176	CD39	A1	TTACCTGGTATCCGT
A0179	CX3CR1	K0124E1	AGTATCGTCTCTGGG
A0180	CD24	ML5	AGATTCCTTCGTGTT
A0181	CD21	Bu32	AACCTAGTAGTTCGG
A0185	CD11a	TS2/4	TATATCCTTGTGAGC
A0187	CD79b (Igβ)	CB3-1	ATTCTTCAACCGAAG
A0189	CD244 (2B4)	C1.7	TCGCTTGGATGGTAG
A0206	CD169 (Sialoadhesin, Siglec-1)	7-239	TACTCAGCGTGTTTG
A0214	integrin β7	FIB504	TCCTTGGATGTACCG
A0215	CD268 (BAFF-R)	11C1	CGAAGTCGATCCGTA
A0216	CD42b	HIP1	TCCTAGTACCGAAGT
A0217	CD54	HA58	CTGATAGACTTGAGT
A0218	CD62P (P-Selectin)	AK4	CCTTCCGTATCCCTT
A0219	CD119 (IFN-γ R α chain)	GIR-208	TGTGTATTCCCTTGT
A0224	TCR α/β	IP26	CGTAACGTAGAGCGA
A0236	Rat IgG1, κ isotype Ctrl	RTK2071	ATCAGATGCCCTCAT
A0237	Rat IgG1, λ Isotype Ctrl	G0114F7	GGGAGCGATTCAACT
A0238	Rat IgG2a, κ Isotype Ctrl	RTK2758	AAGTCAGGTTCGTTT
A0240	Rat IgG2c, κ Isotype Ctrl	RTK4174	TCCAGGCTAGTCATT
A0241	Armenian Hamster IgG Isotype Ctrl	HTK888	CCTGTCATTAAGACT
A0242	CD192 (CCR2)	K036C2	GAGTTCCCTTACCTG
A0246	CD122 (IL-2Rβ)	TU27	TCATTTCCTCCGATT
A0247	CD267 (TACI)	1A1	AGTGATGGAGCGAAC
A0352	FcεRIα	AER-37 (CRA-1)	CTCGTTTCCGTATCG
A0353	CD41	HIP8	ACGTTGTGGCCTTGT
A0355	CD137 (4-1BB)	4B4-1	CAGTAAGTTCGGGAC
A0357	CD43	CD43-10G7	GATTAACCAGCTCAT
A0358	CD163	GHI/61	GCTTCTCCTTCCTTA
A0359	CD83	HB15e	CCACTCATTTCCGGT
A0364	CD13	WM15	TTTCAACGCCCTTTC
A0367	CD2	TS1/8	TACGATTTGTCAGGG
A0368	CD226 (DNAM-1)	11A8	TCTCAGTGTTTGTGG
A0369	CD29	TS2/16	GTATTCCCTCAGTCA
A0370	CD303 (BDCA-2)	201A	GAGATGTCCGAATTT
A0371	CD49b	P1E6-C5	GCTTTCTTCAGTATG
A0372	CD61	VI-PL2	AGGTTGGAGTAGACT

(continued)

DNA_ID	Description	Clone	Barcode
A0373	CD81 (TAPA-1)	5A6	GTATCCTTCCTTGGC
A0383	CD55	JS11	GCTCATTACCCATTA
A0384	IgD	IA6-2	CAGTCTCCGTAGAGT
A0385	CD18	TS1/18	TATTGGGACACTTCT
A0386	CD28	CD28.2	TGAGAACGACCCTAA
A0389	CD38	HIT2	TGTACCCGCTTGTGA
A0390	CD127 (IL-7Rα)	A019D5	GTGTGTTGTCCTATG
A0391	CD45	HI30	TGCAATTACCCGGAT
A0393	CD22	S-HCL-1	GGGTTGTTGTCTTTG
A0394	CD71	CY1G4	CCGTGTTCCTCATTA
A0396	CD26	BA5b	GGTGGCTAGATAATG
A0398	CD115 (CSF-1R)	9-4D2-1E4	AATCACGGTCCTTGT
A0404	CD63	H5C6	GAGATGTCTGCAACT
A0406	CD304 (Neuropilin-1)	12C2	GGACTAAGTTTCGTT
A0407	CD36	5-271	TTCTTTGCCTTGCCA
A0408	CD172a (SIRPα)	15-414	CGTGTTTAACTTGAG
A0419	CD72	3F3	CAGTCGTGGTAGATA
A0420	CD158 (KIR2DL1/S1/S3/S5)	HP-MA4	TATCAACCAACGCTT
A0446	CD93	VIMD2	GCGCTACTTCCTTGA
A0447	CD200 (OX2)	OX-104	CACGTAGACCTTTGC
A0575	CD49a	TS2/7	ACTGATGGACTCAGA
A0576	CD49d	9F10	CCATTCAACTTCCGG
A0577	CD73 (Ecto-5'-nucleotidase)	AD2	CAGTTCCTCAGTTCG
A0579	CD9	HI9a	GAGTCACCAATCTGC
A0581	TCR Vα7.2	3C10	TACGAGCAGTATTCA
A0582	TCR Vδ2	B6	TCAGTCAGATGGTAT
A0586	CD354 (TREM-1)	TREM-26	TAGCCGTTTCCTTTG
A0590	CD305 (LAIR1)	NKTA255	ATTTCCATTCCCTGT
A0591	LOX-1	15C4	ACCCTTTACCGAATA
A0599	CD158e1 (KIR3DL1, NKB1)	DX9	GGACGCTTTCCTTGA
A0817	CD109	W7C5	CACTTAACTCTGGGT
A0822	CD142	NY2	CACTGCCGTCGATTA
A0830	CD319 (CRACC)	162.1	AGTATGCCATGTCTT
A0845	CD99	3B2/TA8	ACCCGTCCCTAAGAA
A0853	CLEC12A	50C1	CATTAGAGTCTGCCA
A0861	CD151 (PETA-3)	50-6	CTTACCTAGTCATTC
A0864	CD352 (NTB-A)	NT-7	AGTTTCCACTCAGGC
A0866	CLEC1B (CLEC2)	AYP1	TGCCAGTATCACGTA
A0867	CD94	DX22	CTTTCCGGTCCTACA
A0868	IgE	MHE-18	GGATGTACCGCGTAT
A0870	CD150 (SLAM)	A12 (7D4)	GTCATTGTATGTCTG
A0871	CD162	KPL-1	ATATGTCAGAGCACC
A0872	CD84	CD84.1.21	CTCCCTAGTTCCTTT
A0894	Ig light chain κ	MHK-49	AGCTCAGCCAGTATG
A0896	CD85j (ILT2)	GHI/75	CCTTGTGAGGCTATG
A0897	CD23	EBVCS-5	TCTGTATAACCGTCT
A0898	Ig light chain λ	MHL-38	CAGCCAGTAAGTCAC
A0902	CD328 (Siglec-7)	6-434	CTTAGCATTTCACTG
A0912	GPR56	CG4	GCCTAGTTTCCGTTT
A0920	CD82	ASL-24	TCCCACTTCCGCTTT
A0923	NKp80	5D12	TATAGTTCCTCTGTG
A0931	CD131	1C1	CTGCATGAGACCAAA

(continued)

DNA_ID	Description	Clone	Barcode
A0935	CD74	LN2	CTGTAGCATTTCCCT
A0940	CD116	4H1	ATGGACAGTTCGTGT
A0941	CD37	M-B371	ACAGTCACTGGGCAA
A0944	CD101 (BB27)	BB27	CTACTTCCCTGTCAA
A1018	HLA-DR, DP, DQ	Tü39	AGCTACGAGCAGTAG
A1046	CD88 (C5aR)	S5/1	GCCGCATGAGAAACA

Supplemental Table 2: Significant surface markers enriched in each cluster (over all other cells) for *in vitro* model. Significance calculated in Seurat using two-sided Wilcoxon test with multiple comparison adjustment using Bonferroni-Hochberg method.

Cluster	Marker	log2(fold change)	Adjusted p value	π-score
C1	CD22	1.40	2.0e-215	301.34
C1	CD72	1.52	1.0e-198	300.64
C1	CD20	1.26	2.5e-218	273.94
C1	CD40	1.17	6.4e-226	263.86
C1	HLA-DR	1.20	2.2e-217	259.29
C1	CD19	1.18	5.6e-217	255.89
C1	CD32	1.14	5.3e-215	245.08
C1	HLA-DR, DP, DQ	1.16	4.1e-212	244.45
C1	CD21	0.95	1.3e-240	227.59
C1	CD86	1.01	9.1e-209	210.48
C1	CD71	0.96	1.0e-204	196.72
C1	CD35	1.06	3.0e-155	164.48
C1	IgD	1.26	3.2e-128	161.24
C1	CD85j (ILT2)	0.78	2.2e-197	153.44
C1	CD39	0.75	1.9e-201	150.80
C1	CD185 (CXCR5)	0.66	1.7e-211	138.28
C1	CD63	0.64	1.2e-167	106.64
C1	CD150 (SLAM)	0.71	4.8e-145	103.18
C1	CD54	0.59	4.7e-174	101.59
C1	CD267 (TACI)	0.62	1.0e-151	93.58
C1	CD79b (Igβ)	0.57	7.7e-140	79.30
C1	CD82	0.36	2.2e-160	57.26
C1	CD119 (IFN-γ R α chain)	0.47	7.4e-116	53.72
C1	CD74	0.35	9.3e-144	50.56
C1	CD45RA	0.38	4.6e-114	42.61
C1	CD196 (CCR6)	0.43	4.0e-92	38.88
C1	CD123	0.43	1.2e-88	37.49
C1	CD73 (Ecto-5'-nucleotidase)	0.46	1.5e-80	36.40
C1	CD37	0.30	5.2e-102	30.09
C1	CD58 (LFA-3)	0.32	2.0e-79	25.23
C1	CD1c	0.29	2.2e-82	24.08
C1	CD69	0.34	3.5e-69	23.37
C1	CD319 (CRACC)	0.31	2.7e-71	21.63
C1	IgM	0.28	5.0e-62	17.24
C1	CD183 (CXCR3)	0.29	2.4e-56	16.31
C1	CD272 (BTLA)	0.26	6.5e-51	12.80
C1	CD83	0.29	6.2e-37	10.57
C10	CD244 (2B4)	1.05	4.6e-218	227.73
C10	KLRG1 (MAFA)	1.11	6.4e-173	190.52
C10	CD57	1.23	7.3e-134	163.63
C10	GPR56	0.84	4.6e-170	142.86
C10	CD195 (CCR5)	0.90	3.1e-145	129.71
C10	CD49a	0.94	3.3e-130	121.32
C10	HLA-DR, DP, DQ	0.62	1.9e-111	69.05
C10	CD29	0.43	6.2e-136	58.01
C10	HLA-DR	0.52	2.2e-101	52.16
C10	CD319 (CRACC)	0.37	3.8e-97	36.04
C10	CD18	0.27	3.4e-98	26.36
C10	CD49d	0.29	1.2e-86	25.06
C10	CD279 (PD-1)	0.40	5.7e-59	23.28
C10	CD151 (PETA-3)	0.34	4.3e-57	19.10
C10	CD226 (DNAM-1)	0.25	9.8e-57	14.24
C10	TIGIT (VSTM3)	0.39	2.0e-33	12.80
C10	CD223 (LAG-3)	0.31	1.7e-33	10.21

(continued)

Cluster	Marker	log2(fold change)	Adjusted p value	π-score
C2	CD112 (Nectin-2)	0.50	1.4e-23	11.48
C2	CD74	0.42	1.6e-26	10.75
C2	CX3CR1	0.44	7.8e-22	9.19
C2	CD79b (Igβ)	0.43	1.9e-20	8.46
C2	CD23	0.40	6.8e-20	7.70
C2	LOX-1	0.37	1.0e-18	6.73
C2	CD134 (OX40)	0.39	1.6e-16	6.23
C2	CD116	0.28	3.0e-21	5.65
C2	CD24	0.32	5.1e-18	5.47
C2	CD303 (BDCA-2)	0.29	3.8e-16	4.55
C2	CD314 (NKG2D)	0.26	8.6e-17	4.15
C2	CD83	0.30	2.2e-12	3.54
C2	IgE	0.27	2.9e-13	3.42
C2	CD163	0.31	1.8e-09	2.75
C2	CD141 (Thrombomodulin)	0.28	1.5e-09	2.43
C2	CD275 (B7-H2, B7-RP1, ICOSL)	0.25	3.4e-10	2.38
C2	CD123	0.29	7.1e-04	0.90
C3	TIGIT (VSTM3)	0.79	3.6e-61	47.92
C3	CD25	0.79	2.5e-57	44.44
C3	CD95 (Fas)	0.27	2.8e-43	11.58
C3	CD49f	0.37	2.7e-25	9.16
C3	CD194 (CCR4)	0.32	1.2e-20	6.34
C4	CD49b	0.61	5.9e-159	96.86
C4	CD195 (CCR5)	0.56	7.3e-136	76.22
C4	CD161	0.69	8.3e-108	73.66
C4	CD150 (SLAM)	0.41	1.7e-141	57.48
C4	CD194 (CCR4)	0.36	4.8e-96	34.56
C4	CD49a	0.42	2.8e-81	33.52
C4	CD226 (DNAM-1)	0.26	4.2e-126	32.90
C4	CD25	0.33	1.2e-96	31.57
C4	CD58 (LFA-3)	0.26	8.3e-119	31.27
C4	CD279 (PD-1)	0.30	8.3e-93	27.92
C4	CD151 (PETA-3)	0.26	4.9e-74	19.24
C4	CD69	0.39	2.2e-46	17.62
C5	CD49b	0.56	5.3e-182	102.14
C5	CD194 (CCR4)	0.42	1.1e-163	67.84
C5	CD161	0.56	7.0e-92	51.28
C5	CD29	0.27	7.6e-187	50.77
C5	CD226 (DNAM-1)	0.26	2.7e-192	50.35
C5	CD58 (LFA-3)	0.27	1.9e-173	47.04
C5	CD150 (SLAM)	0.32	2.3e-145	46.34
C5	CD195 (CCR5)	0.42	1.1e-96	40.04
C5	CD49a	0.33	1.6e-79	26.33
C6	CD55	0.44	0.0e+00	Inf
C6	CD45RA	0.25	6.5e-248	62.04
C7	CD38	0.33	1.8e-177	58.69
C7	CD7	0.27	4.0e-112	30.17
C7	CD31	0.32	5.0e-59	18.57
C8	CD38	0.38	0.0e+00	Inf
C8	CD7	0.35	0.0e+00	Inf
C8	CD27	0.27	0.0e+00	Inf
C8	CD31	0.45	1.9e-203	91.10
C8	CD101 (BB27)	0.31	3.6e-165	50.61
C8	CD183 (CXCR3)	0.25	6.1e-159	40.20
C8	CD9	0.29	1.0e-50	14.26

Supplemental Table 3: Significant surface markers enriched in HIV+ versus HIV- cells for *in vitro* model. Test performed using the DESeq2 pseudobulk method in Seurat (two-tailed with multiple comparison adjustment using Bonferroni-Hochberg method).

Subset	Cluster	Marker	log2(fold change)	Adjusted p value	π-score
CD4+	HIV-	CD31	-1.06	1.0e-158	-167.94
CD4+	HIV-	CD62L	-0.52	9.1e-265	-136.14
CD4+	HIV-	CD55	-0.52	1.0e-178	-91.74
CD4+	HIV-	CD27	-0.37	1.8e-241	-89.81
CD4+	HIV-	CD7	-0.38	5.9e-123	-46.54
CD4+	HIV-	CD9	-0.52	8.2e-45	-23.03
CD4+	HIV-	CD45RA	-0.22	1.9e-89	-19.14
CD4+	HIV-	CD38	-0.27	1.4e-44	-12.02
CD4+	HIV-	CD3	-0.06	7.5e-83	-4.54
CD4+	HIV-	TCR α/β	-0.06	8.6e-72	-4.20
CD4+	HIV-	CD127 (IL-7Rα)	-0.09	4.5e-41	-3.54
CD4+	HIV-	CD272 (BTLA)	-0.11	1.3e-25	-2.69
CD4+	HIV+	CD195 (CCR5)	2.08	2.2e-308	640.57
CD4+	HIV+	CD150 (SLAM)	1.64	2.2e-308	504.37
CD4+	HIV+	CD279 (PD-1)	1.36	1.3e-228	309.43
CD4+	HIV+	CD49a	1.68	1.7e-172	288.33
CD4+	HIV+	CD161	1.94	7.5e-145	279.09
CD4+	HIV+	CD2	0.87	2.2e-308	267.60
CD4+	HIV+	CD95 (Fas)	0.97	5.6e-260	251.16
CD4+	HIV+	CD49b	1.61	1.6e-154	247.25
CD4+	HIV+	CD99	0.79	1.4e-235	185.04
CD4+	HIV+	CD29	1.03	1.1e-173	177.44
CD4+	HIV+	KLRG1 (MAFA)	1.39	4.4e-115	158.63
CD4+	HIV+	GPR56	1.34	1.4e-113	151.04
CD4+	HIV+	HLA-DR, DP, DQ	1.12	2.3e-134	149.85
CD4+	HIV+	CD151 (PETA-3)	1.04	1.3e-141	146.42
CD4+	HIV+	CD226 (DNAM-1)	0.84	3.9e-151	126.37
CD4+	HIV+	CD58 (LFA-3)	0.89	1.4e-121	107.82
CD4+	HIV+	CD244 (2B4)	1.21	4.7e-89	107.21
CD4+	HIV+	CD69	1.37	1.1e-74	101.26
CD4+	HIV+	HLA-DR	0.94	2.2e-100	93.74
CD4+	HIV+	CD11a	0.60	3.4e-132	79.11
CD4+	HIV+	CD196 (CCR6)	0.99	2.2e-66	65.06
CD4+	HIV+	CD49d	0.77	4.1e-83	63.34
CD4+	HIV+	CD278 (ICOS)	0.66	1.2e-95	62.58
CD4+	HIV+	CD45RO	0.65	1.1e-85	55.12
CD4+	HIV+	CD25	0.85	1.2e-53	45.13
CD4+	HIV+	CD192 (CCR2)	0.91	4.4e-49	43.98
CD4+	HIV+	CD18	0.53	1.9e-75	39.68
CD4+	HIV+	CD54	0.61	2.0e-65	39.15
CD4+	HIV+	CD26	0.70	1.9e-53	36.72
CD4+	HIV+	CD194 (CCR4)	0.80	3.7e-44	34.62
CD4+	HIV+	CD84	0.69	3.4e-49	33.30
CD4+	HIV+	CD146	0.84	8.1e-40	32.93
CD4+	HIV+	CD82	0.54	5.5e-54	28.76
CD4+	HIV+	CD39	0.60	9.0e-45	26.33
CD4+	HIV+	CD119 (IFN-γ R α chain)	0.60	1.7e-35	20.78
CD4+	HIV+	CD81 (TAPA-1)	0.52	9.3e-24	11.99
CD4+	HIV+	CD305 (LAIR1)	0.52	4.6e-22	11.02
CD4+	HIV+	CD352 (NTB-A)	0.43	3.0e-20	8.36
CD4+	HIV+	CD73 (Ecto-5'-nucleotidase)	0.63	1.3e-13	8.05
CD4+	HIV+	CD45	0.34	4.2e-22	7.27
CD4+	HIV+	CD48	0.38	4.9e-19	7.05
CD4+	HIV+	HLA-A,B,C	0.43	2.2e-16	6.79

(continued)

Subset	Cluster	Marker	log2(fold change)	Adjusted p value	π-score
CD4+	HIV+	integrin β7	0.47	3.9e-08	3.50
CD4+	HIV+	CD44	0.35	2.0e-10	3.36
CD4+	HIV+	CD183 (CXCR3)	0.39	3.6e-07	2.50
CD4+	HIV+	CD52	0.12	5.3e-19	2.25
CD4+	HIV+	CD43	0.30	3.3e-07	1.94
CD4+	HIV+	CD47	0.01	1.8e-102	1.17
CD4+	HIV+	CD101 (BB27)	0.37	6.0e-03	0.81
CD4+	HIV+	CD19	0.12	2.6e-07	0.81
activated/late diff CD4+	HIV-	CD62L	-0.38	1.0e-54	-20.54
activated/late diff CD4+	HIV-	CD127 (IL-7Rα)	-0.28	1.7e-20	-5.57
activated/late diff CD4+	HIV-	CD49f	-0.31	4.4e-14	-4.15
activated/late diff CD4+	HIV-	CD185 (CXCR5)	-0.26	8.6e-13	-3.09
activated/late diff CD4+	HIV-	CD194 (CCR4)	-0.18	3.1e-08	-1.33
activated/late diff CD4+	HIV-	CD27	-0.06	3.7e-14	-0.77
activated/late diff CD4+	HIV-	TIGIT (VSTM3)	-0.14	7.2e-06	-0.70
activated/late diff CD4+	HIV-	CD31	-0.10	2.6e-04	-0.37
activated/late diff CD4+	HIV-	CD55	-0.02	3.1e-05	-0.07
activated/late diff CD4+	HIV-	CD7	-0.02	8.7e-03	-0.04
activated/late diff CD4+	HIV-	CD5	-0.01	1.0e-03	-0.02
activated/late diff CD4+	HIV-	CD162	0.00	1.1e-03	-0.01
activated/late diff CD4+	HIV+	CD195 (CCR5)	0.90	2.1e-32	28.34
activated/late diff CD4+	HIV+	CD150 (SLAM)	0.79	1.9e-35	27.31
activated/late diff CD4+	HIV+	CD2	0.41	4.4e-36	14.40
activated/late diff CD4+	HIV+	GPR56	0.86	7.3e-15	12.12
activated/late diff CD4+	HIV+	CD279 (PD-1)	0.66	1.1e-18	11.79
activated/late diff CD4+	HIV+	CD95 (Fas)	0.33	3.8e-16	5.13
activated/late diff CD4+	HIV+	CD119 (IFN-γ R α chain)	0.46	2.5e-10	4.46
activated/late diff CD4+	HIV+	CD26	0.41	2.5e-06	2.32
activated/late diff CD4+	HIV+	HLA-DR, DP, DQ	0.46	2.3e-05	2.13
activated/late diff CD4+	HIV+	CD244 (2B4)	0.57	2.8e-04	2.04
activated/late diff CD4+	HIV+	CD11a	0.27	5.9e-08	1.97
activated/late diff CD4+	HIV+	CD305 (LAIR1)	0.42	2.3e-05	1.94
activated/late diff CD4+	HIV+	CD146	0.55	4.1e-04	1.88
activated/late diff CD4+	HIV+	CD161	0.59	2.1e-03	1.57
activated/late diff CD4+	HIV+	CD73 (Ecto-5’-nucleotidase)	0.56	1.9e-03	1.53
activated/late diff CD4+	HIV+	HLA-DR	0.43	3.4e-04	1.49
activated/late diff CD4+	HIV+	CD54	0.29	9.6e-06	1.43
activated/late diff CD4+	HIV+	CD151 (PETA-3)	0.38	1.6e-03	1.07
activated/late diff CD4+	HIV+	CD196 (CCR6)	0.48	6.5e-03	1.06
activated/late diff CD4+	HIV+	CD192 (CCR2)	0.52	1.7e-02	0.92
activated/late diff CD4+	HIV+	CD48	0.23	2.5e-04	0.82
activated/late diff CD4+	HIV+	CD25	0.30	2.1e-03	0.81
activated/late diff CD4+	HIV+	CD49b	0.38	9.3e-03	0.77
activated/late diff CD4+	HIV+	KLRG1 (MAFA)	0.50	3.4e-02	0.74
activated/late diff CD4+	HIV+	CD49a	0.43	4.5e-02	0.58
activated/late diff CD4+	HIV+	CD101 (BB27)	0.39	4.1e-02	0.53
activated/late diff CD4+	HIV+	CD99	0.22	5.1e-03	0.50
activated/late diff CD4+	HIV+	CD47	0.05	5.9e-07	0.30
activated/late diff CD4+	HIV+	CD52	0.07	3.0e-03	0.17
activated/late diff CD4+	HIV+	CD82	0.07	1.7e-02	0.13
early diff CD4+	HIV-	CD62L	-0.27	7.0e-31	-8.17
early diff CD4+	HIV-	CD55	-0.24	1.3e-19	-4.57
early diff CD4+	HIV-	CD127 (IL-7Rα)	-0.09	4.5e-14	-1.21
early diff CD4+	HIV-	CD162	-0.10	1.7e-10	-0.96
early diff CD4+	HIV-	CD45RA	-0.08	1.2e-10	-0.77
early diff CD4+	HIV+	CD278 (ICOS)	0.57	3.0e-17	9.46

(continued)

Subset	Cluster	Marker	log2(fold change)	Adjusted p value	π-score
early diff CD4+	HIV+	CD25	0.79	2.5e-12	9.21
early diff CD4+	HIV+	CD183 (CXCR3)	0.77	8.0e-10	7.00
early diff CD4+	HIV+	CD11a	0.55	1.1e-12	6.53
early diff CD4+	HIV+	CD49b	0.67	1.7e-09	5.87
early diff CD4+	HIV+	CD2	0.50	3.4e-09	4.24
early diff CD4+	HIV+	CD38	0.60	5.4e-07	3.74
early diff CD4+	HIV+	CD151 (PETA-3)	0.60	1.4e-06	3.54
early diff CD4+	HIV+	CD305 (LAIR1)	0.57	8.3e-06	2.89
early diff CD4+	HIV+	CD71	0.52	2.5e-04	1.87
early diff CD4+	HIV+	CD18	0.45	1.1e-04	1.80
early diff CD4+	HIV+	CD47	0.10	4.5e-17	1.69
early diff CD4+	HIV+	CD101 (BB27)	0.57	1.6e-03	1.61
early diff CD4+	HIV+	HLA-A,B,C	0.18	5.3e-07	1.10
early diff CD4+	HIV+	CD95 (Fas)	0.42	5.6e-03	0.95
early diff CD4+	HIV+	CD4	0.42	1.1e-02	0.82
early diff CD4+	HIV+	CD19	0.12	3.9e-02	0.16
early diff CD4+	HIV+	CD49f	0.04	3.0e-03	0.10

Supplemental Table 4: Differential peak list for activated CD4+ cells between HIV+ and HIV- for *in vitro* model.

Subset	Chr	Start	End	Log2FC	FDR	π-score	In Gene	Nearest TSS	Nearest TSS Dist
HIV-	chrX	133763071	133763571	-5.76	2.0e-02	-9.73	GPC3	GPC3	222324
HIV-	chr11	128840083	128840583	-2.32	3.4e-04	-8.04	KCNJ1	KCNJ1	26790
HIV-	chr3	120344302	120344802	-1.62	4.3e-04	-5.47	LRRC58	LRRC58	4537
HIV-	chr16	29013010	29013510	-1.51	4.3e-04	-5.08	MIR3680-1	LAT	28184
HIV-	chr5	75078009	75078509	-2.44	9.5e-03	-4.94	ANKRD31	LINC01336	-25166
HIV-	chr17	62164986	62165486	-3.33	4.4e-02	-4.51	-	MED13	-99704
HIV-	chr13	73984895	73985395	-1.91	7.8e-03	-4.03	KLF12	KLF12	148510
HIV-	chr7	127322273	127322773	-1.49	2.1e-03	-4.00	-	GRM8	-68979
HIV-	chr6	149366167	149366667	-1.44	1.8e-03	-3.93	TAB2	SUMO4	-33560
HIV-	chr1	87045694	87046194	-2.48	2.7e-02	-3.89	HS2ST1, LINC01140	LINC01140	52685
HIV-	chr5	109921610	109922110	-1.41	2.0e-03	-3.82	-	LINC01848	38428
HIV-	chr5	39186867	39187367	-1.84	8.4e-03	-3.82	FYB1	FYB1	87161
HIV-	chr10	3800532	3801032	-2.74	4.7e-02	-3.65	-	KLF6	-15251
HIV-	chr1	39653445	39653945	-1.90	1.7e-02	-3.35	-	HEYL	-13500
HIV-	chr3	10263496	10263996	-2.19	3.0e-02	-3.34	TATDN2	TATDN2	15473
HIV-	chr8	120472266	120472766	-2.41	4.5e-02	-3.25	MTBP	MRPL13	-26864
HIV-	chr7	1925895	1926395	-1.53	7.8e-03	-3.23	MAD1L1	MIR4655	-81642
HIV-	chrX	136674455	136674955	-1.51	7.8e-03	-3.18	ARHGEF6	CD40LG	26262
HIV-	chr12	64494944	64495444	-1.89	2.1e-02	-3.17	TBK1	TBK1	43064
HIV-	chr15	44560876	44561376	-1.87	2.1e-02	-3.15	EIF3J	EIF3J	23819
HIV-	chr8	120775213	120775713	-2.19	3.8e-02	-3.13	SNTB1, LOC101927543	LOC101927543	13960
HIV-	chr11	14463556	14464056	-2.33	4.7e-02	-3.10	COPB1	COPB1	35971
HIV-	chr6	37289865	37290365	-2.29	4.6e-02	-3.06	TBC1D22B	TMEM217	-31710
HIV-	chr15	88904581	88905081	-1.01	2.0e-03	-2.72	MFGE8	MFGE8	8330
HIV-	chr3	153643435	153643935	-1.59	2.1e-02	-2.68	-	C3orf79	158940
HIV-	chr16	29827058	29827558	-1.85	4.4e-02	-2.52	BOLA2, SLX1A, SLX1A-SULT1A3, SULT1A4, LOC388242, PAGR1, MVP	MVP	6664
HIV-	chr2	203987082	203987582	-1.65	4.4e-02	-2.24	-	ICOS	50334
HIV-	chr3	187027350	187027850	-1.03	7.8e-03	-2.18	ST6GAL1	ST6GAL1	96865
HIV-	chr10	118732697	118733197	-1.59	4.8e-02	-2.09	CACUL1	CACUL1	22052
HIV-	chr3	118955786	118956286	-1.29	4.1e-02	-1.78	IGSF11	IGSF11-AS1	12713
HIV-	chr13	49371836	49372336	-1.12	3.1e-02	-1.70	CAB39L	CAB39L	71790
HIV-	chr14	32102444	32102944	-1.21	4.5e-02	-1.63	ARHGAP5	ARHGAP5-AS1	-25651
HIV-	chr10	43332102	43332602	-1.16	4.0e-02	-1.62	-	FXYD4	-39040
HIV-	chr1	100927241	100927741	-0.91	3.0e-02	-1.38	SLC30A7	SLC30A7	31165

(continued)

Subset	Chr	Start	End	Log2FC	FDR	π-score	In Gene	Nearest TSS	Nearest TSS Dist
HIV-	chr17	66331088	66331588	-0.94	3.4e-02	-1.38	PRKCA	PRKCA	28452
HIV-	chr14	89903512	89904012	-1.01	4.7e-02	-1.34	FOXN3, EFCAB11	FOXN3	50647
HIV-	chr13	51825061	51825561	-0.85	2.7e-02	-1.33	TMEM272	TMEM272	19614
HIV-	chr16	27235313	27235813	-0.99	4.6e-02	-1.32	MIR3680-1, NSMCE1	KDM8	31818
HIV-	chr10	110225646	110226146	-0.83	2.6e-02	-1.32	MXI1	MXI1	18041
HIV-	chr13	40485590	40486090	-0.61	1.5e-02	-1.10	LINC00598	LINC00598	49717
HIV+	chr1	221829595	221830095	1.39	3.9e-05	6.15	LINC01655	LINC01655	10571
HIV+	chr9	73869213	73869713	2.09	1.4e-03	5.99	-	LOC101927358	-501818
HIV+	chr6	117465645	117466145	2.53	6.7e-03	5.50	GOPC, DCBLD1	DCBLD1	11828
HIV+	chr14	68719698	68720198	1.16	3.5e-05	5.17	-	ZFP36L1	76055
HIV+	chr8	72000175	72000675	1.52	1.4e-03	4.34	MSC-AS1	TRPA1	74942
HIV+	chr14	59275611	59276111	1.76	5.1e-03	4.05	DAAM1	DAAM1	86965
HIV+	chr15	101199696	101200196	1.15	3.3e-04	4.00	CHSY1	CHSY1	51736
HIV+	chr9	75148484	75148984	2.03	1.1e-02	3.99	-	OSTF1	59941
HIV+	chr15	90412628	90413128	2.94	4.4e-02	3.98	IQGAP1	IQGAP1	24410
HIV+	chr6	13747556	13748056	1.10	4.3e-04	3.70	-	RANBP9	-35992
HIV+	chr2	101223824	101224324	1.52	5.4e-03	3.44	TBC1D8	CNOT11	-28478
HIV+	chr2	136362680	136363180	1.86	1.5e-02	3.38	-	CXCR4	-244515
HIV+	chr3	71447689	71448189	2.51	4.6e-02	3.36	FOXP1	MIR1284	93900
HIV+	chr9	86348418	86348918	1.66	9.7e-03	3.35	TUT7	TUT7	5536
HIV+	chr7	23595009	23595509	2.50	4.7e-02	3.32	-	CCDC126	-1870
HIV+	chr11	14288180	14288680	1.57	9.1e-03	3.22	RRAS2	RRAS2	75826
HIV+	chr3	71392986	71393486	1.90	2.0e-02	3.22	FOXP1	FOXP1-AS1	103217
HIV+	chr17	3795234	3795734	1.80	2.0e-02	3.04	ITGAE	ITGAE	5509
HIV+	chr1	25099439	25099939	2.11	3.8e-02	3.00	-	MIR4425	75936
HIV+	chr11	43316418	43316918	2.09	3.8e-02	2.97	API5	API5	4455
HIV+	chr13	27131122	27131622	0.85	3.8e-04	2.89	USP12	USP12-AS1	-31233
HIV+	chr5	72194799	72195299	2.07	4.0e-02	2.88	MAP1B	MIR4803	25332
HIV+	chr4	26076133	26076633	1.52	1.5e-02	2.75	-	RBPJ	-86822
HIV+	chr16	82660155	82660655	2.05	4.8e-02	2.70	CDH13	MIR8058	-28276
HIV+	chr15	38610792	38611292	1.30	9.1e-03	2.66	-	RASGRP1	-45217
HIV+	chr8	133152859	133153359	1.20	7.0e-03	2.58	-	WISP1	-37680
HIV+	chr8	28365700	28366200	1.93	4.8e-02	2.53	ZNF395	PNOC	48714
HIV+	chr10	22505996	22506496	1.85	4.5e-02	2.48	-	SPAG6	160551
HIV+	chr11	34235166	34235666	1.43	2.0e-02	2.41	ABTB2	ABTB2	122342
HIV+	chr4	121256332	121256832	1.18	9.5e-03	2.39	-	TNIP3	-28866
HIV+	chr16	23504680	23505180	1.09	6.7e-03	2.37	MIR3680-1, GGA2	GGA2	16815
HIV+	chr5	127785555	127786055	1.07	8.4e-03	2.22	CCDC192	CCDC192	82165
HIV+	chr8	97980061	97980561	1.27	2.0e-02	2.14	MATN2	SNORA72	61656
HIV+	chr10	22597125	22597625	1.60	4.7e-02	2.13	PIP4K2A	PIP4K2A	116930

(continued)

Subset	Chr	Start	End	Log2FC	FDR	π-score	In Gene	Nearest TSS	Nearest TSS Dist
HIV+	chr2	65232542	65233042	1.24	2.0e-02	2.10	ACTR2	ACTR2	4789
HIV+	chr11	128551675	128552175	1.22	2.2e-02	2.03	ETS1	ETS1-AS1	25533
HIV+	chr13	50245013	50245513	1.42	3.9e-02	2.01	DLEU1	DLEU2	-119293
HIV+	chr3	46369194	46369694	1.43	4.1e-02	1.97	LOC102724297	CCR5	-1160
HIV+	chr10	88270827	88271327	1.03	1.2e-02	1.96	-	RNLS	313203
HIV+	chr6	108921393	108921893	1.19	2.4e-02	1.94	ARMC2	ARMC2-AS1	2210
HIV+	chr1	32263726	32264226	1.42	4.5e-02	1.91	LCK	LCK	12487
HIV+	chr8	100493218	100493718	1.33	4.7e-02	1.76	-	ANKRD46	66066
HIV+	chr4	101039328	101039828	1.24	4.1e-02	1.73	PPP3CA	EMCN	-159202
HIV+	chr2	178514591	178515091	0.78	6.4e-03	1.71	PLEKHA3	TTN-AS1	-6092
HIV+	chr3	155862523	155863023	1.29	4.8e-02	1.70	-	GMPS	-7513
HIV+	chr14	74573941	74574441	1.08	4.3e-02	1.48	LTBP2	LTBP2	37937
HIV+	chr22	39961397	39961897	0.87	2.0e-02	1.47	GRAP2	FAM83F	-33052
HIV+	chr13	76991309	76991809	0.65	1.3e-02	1.21	CLN5	CLN5	649
HIV+	chr8	66467771	66468271	0.76	4.1e-02	1.06	ADHFE1, VXN	VXN	7768

Supplemental Table 5: Significant surface markers enriched in each cluster (over all other cells) during chronic infection. Significance calculated in Seurat using two-sided Wilcoxon test with multiple comparison adjustment using Bonferroni-Hochberg method.

Cluster	Marker	log2(fold change)	Adjusted p value	π-score
C1	CX3CR1	0.62	4.3e-226	138.97
C1	CD275 (B7-H2, B7-RP1, ICOSL)	0.57	1.6e-235	133.74
C1	CD303 (BDCA-2)	0.52	2.6e-220	115.20
C1	IgE	0.53	2.9e-205	109.34
C1	LOX-1	0.51	2.4e-207	105.69
C1	CD79b (Igβ)	0.55	9.5e-188	102.25
C1	CD74	0.50	1.5e-192	95.36
C1	CD83	0.51	1.1e-184	93.93
C1	NKp80	0.47	9.0e-194	91.56
C1	CD137 (4-1BB)	0.43	1.1e-210	89.65
C1	CD142	0.42	4.6e-197	82.21
C1	CD93	0.45	2.1e-180	81.08
C1	CD169 (Sialoadhesin, Siglec-1)	0.42	1.8e-185	77.71
C1	CD23	0.46	2.6e-163	74.28
C1	CD163	0.47	1.8e-155	72.27
C1	CD192 (CCR2)	0.44	2.7e-162	71.81
C1	CD134 (OX40)	0.43	1.5e-164	71.25
C1	CD24	0.42	1.8e-160	67.51
C1	CD112 (Nectin-2)	0.41	2.3e-166	67.17
C1	CD154	0.40	4.2e-153	61.51
C1	CD158e1 (KIR3DL1, NKB1)	0.33	3.5e-188	61.28
C1	CD42b	0.30	5.7e-202	59.72
C1	CD45RA	0.39	2.5e-152	58.99
C1	CD223 (LAG-3)	0.41	2.5e-142	58.28
C1	CD116	0.37	1.7e-156	57.63
C1	CD109	0.36	5.9e-152	54.43
C1	FcεRIα	0.32	4.6e-161	51.11
C1	CD85j (ILT2)	0.36	2.7e-141	50.70
C1	CD158 (KIR2DL1/S1/S3/S5)	0.29	1.4e-172	50.35
C1	CD196 (CCR6)	0.37	1.1e-109	40.72
C1	CD14	0.29	2.4e-142	40.51
C1	CD314 (NKG2D)	0.37	9.2e-108	39.46
C1	Ig light chain λ	0.30	1.7e-128	38.92
C1	CD115 (CSF-1R)	0.31	4.8e-119	37.04
C1	KLRG1 (MAFA)	0.33	1.6e-110	36.20
C1	CD13	0.28	1.9e-127	35.76
C1	CD141 (Thrombomodulin)	0.29	6.2e-123	35.06
C1	Ig light chain κ	0.28	4.9e-105	29.01
C1	Podoplanin	0.29	1.1e-95	27.21
C1	CD152 (CTLA-4)	0.26	1.1e-104	26.83
C1	CD122 (IL-2Rβ)	0.28	1.3e-77	21.83
C1	CD150 (SLAM)	0.27	3.0e-78	21.23
C10	CD9	1.50	2.2e-68	101.61
C10	CD38	1.23	2.7e-83	101.21
C10	CD19	1.00	3.9e-79	78.25
C10	CD319 (CRACC)	0.85	1.0e-84	71.54
C10	CD39	0.94	1.5e-64	59.97
C10	CD63	0.94	1.5e-57	53.35
C10	CD21	0.91	2.9e-58	52.55
C10	CD31	0.89	2.6e-59	52.09
C10	CD32	0.96	1.9e-51	48.71
C10	CD54	0.77	4.0e-56	42.44
C10	CD22	0.65	2.7e-46	29.62
C10	CD72	0.66	4.1e-40	26.05

(continued)

Cluster	Marker	log2(fold change)	Adjusted p value	π-score
C10	HLA-DR, DP, DQ	0.73	1.6e-33	23.89
C10	CD55	0.61	9.5e-38	22.74
C10	HLA-DR	0.61	5.2e-34	20.30
C10	CD85j (ILT2)	0.46	9.1e-33	14.86
C10	CD40	0.40	1.9e-28	11.22
C10	CD71	0.46	4.2e-24	10.79
C10	CD35	0.41	3.5e-19	7.58
C10	CD81 (TAPA-1)	0.29	7.9e-18	5.01
C10	CD150 (SLAM)	0.25	5.5e-09	2.10
C11	CD72	1.33	5.4e-166	220.51
C11	CD22	1.28	2.0e-165	211.01
C11	CD35	1.19	8.5e-146	173.19
C11	CD21	1.23	3.1e-139	170.49
C11	CD19	1.03	2.5e-152	156.52
C11	CD32	1.17	1.2e-126	147.83
C11	CD20	0.91	1.4e-155	140.92
C11	CD37	0.79	3.5e-136	106.37
C11	CD40	0.73	2.6e-132	96.03
C11	HLA-DR, DP, DQ	0.83	6.7e-114	93.44
C11	HLA-DR	0.70	4.9e-123	85.04
C11	IgM	0.82	1.2e-101	82.69
C11	CD73 (Ecto-5’-nucleotidase)	0.87	2.4e-84	72.90
C11	CD31	0.56	1.9e-84	46.71
C11	CD196 (CCR6)	0.50	1.9e-74	36.81
C11	CD55	0.52	2.4e-70	35.99
C11	CD85j (ILT2)	0.47	4.2e-75	34.59
C11	CD63	0.46	2.2e-67	30.89
C11	CD54	0.44	5.5e-71	30.69
C11	CD119 (IFN-γ R α chain)	0.38	1.3e-62	23.38
C11	CD24	0.45	9.5e-51	22.64
C11	CD49d	0.40	3.5e-53	21.09
C11	Ig light chain λ	0.50	3.5e-36	17.65
C11	CD74	0.35	5.4e-48	16.46
C11	Ig light chain κ	0.39	1.9e-42	16.38
C11	CD1c	0.35	5.3e-48	16.35
C11	CD82	0.29	3.4e-45	12.86
C11	CD267 (TACI)	0.32	1.9e-39	12.30
C11	Podoplanin	0.33	3.6e-34	10.97
C11	CD83	0.35	2.1e-31	10.73
C11	CD23	0.36	3.0e-28	9.79
C11	CD39	0.30	1.1e-31	9.26
C11	CD305 (LAIR1)	0.32	1.6e-15	4.80
C11	CD9	0.26	1.5e-12	3.04
C2	CD200 (OX2)	1.09	0.0e+00	Inf
C2	CD57	1.06	0.0e+00	Inf
C2	CD279 (PD-1)	0.82	0.0e+00	Inf
C2	CD69	0.78	0.0e+00	Inf
C2	CD71	0.71	0.0e+00	Inf
C2	TIGIT (VSTM3)	0.67	0.0e+00	Inf
C2	CD278 (ICOS)	0.64	0.0e+00	Inf
C2	CD185 (CXCR5)	0.61	0.0e+00	Inf
C2	CD272 (BTLA)	0.59	0.0e+00	Inf
C2	CD11a	0.37	0.0e+00	Inf
C2	CD151 (PETA-3)	0.45	6.8e-305	138.39
C2	CD27	0.40	3.4e-251	101.30
C2	CD18	0.37	8.3e-255	93.60

(continued)

Cluster	Marker	log2(fold change)	Adjusted p value	π-score
C2	CD95 (Fas)	0.28	1.2e-297	83.36
C2	CD45	0.29	1.4e-256	73.97
C2	CD45RO	0.27	7.2e-241	64.65
C3	CD95 (Fas)	0.31	2.7e-88	26.87
C3	CD195 (CCR5)	0.55	1.9e-47	25.52
C3	CD25	0.43	8.8e-58	24.62
C3	CD162	0.38	4.0e-48	18.15
C3	CD38	0.36	1.9e-49	17.39
C3	CD71	0.41	5.0e-41	16.66
C3	CD278 (ICOS)	0.34	4.4e-46	15.52
C3	CD39	0.42	5.7e-37	15.33
C3	HLA-DR, DP, DQ	0.29	8.4e-43	12.13
C3	CD101 (BB27)	0.38	3.5e-22	8.15
C3	CD49d	0.26	3.7e-20	4.98
C3	CD8	0.30	3.4e-10	2.81
C4	CD49f	0.41	7.5e-44	17.58
C4	CD127 (IL-7Rα)	0.39	3.5e-43	16.64
C4	CD29	0.29	4.4e-38	10.92
C5	CD7	0.54	0.0e+00	Inf
C5	CD99	0.46	0.0e+00	Inf
C5	CD5	0.44	0.0e+00	Inf
C5	CD48	0.42	0.0e+00	Inf
C5	CD4	0.36	0.0e+00	Inf
C5	CD47	0.33	0.0e+00	Inf
C5	CD3	0.30	0.0e+00	Inf
C5	CD49f	0.50	7.9e-280	139.01
C5	CD127 (IL-7Rα)	0.46	2.2e-254	117.40
C5	CD62L	0.41	4.7e-251	103.77
C5	CD44	0.32	1.0e-278	88.65
C5	CD69	0.43	1.9e-166	71.55
C5	HLA-A,B,C	0.27	9.4e-251	68.41
C5	CD45	0.29	5.9e-231	67.73
C5	CD52	0.28	3.7e-234	66.02
C5	CD2	0.28	6.5e-229	64.67
C5	CD29	0.31	2.4e-172	53.89
C5	CD27	0.33	3.8e-164	53.36
C5	CD162	0.30	1.3e-157	47.68
C5	CD55	0.32	5.4e-142	45.65
C5	CD305 (LAIR1)	0.31	1.6e-103	32.14
C5	CD49a	0.39	5.5e-60	23.33
C6	CX3CR1	0.61	1.4e-241	147.28
C6	CD303 (BDCA-2)	0.54	1.3e-228	124.11
C6	CD275 (B7-H2, B7-RP1, ICOSL)	0.53	8.6e-220	116.31
C6	IgE	0.53	8.6e-209	111.07
C6	CD83	0.54	1.8e-203	108.95
C6	CD79b (Igβ)	0.50	2.2e-205	103.05
C6	CD24	0.52	8.0e-187	97.40
C6	CD192 (CCR2)	0.49	4.4e-198	96.54
C6	LOX-1	0.48	2.8e-196	94.37
C6	CD169 (Sialoadhesin, Siglec-1)	0.45	1.7e-202	91.08
C6	CD74	0.43	7.0e-207	88.07
C6	NKp80	0.47	8.4e-189	87.53
C6	CD115 (CSF-1R)	0.45	6.4e-195	87.20
C6	CD141 (Thrombomodulin)	0.42	1.4e-206	86.29
C6	CD223 (LAG-3)	0.49	2.9e-172	84.63
C6	CD23	0.45	1.0e-170	76.74

(continued)

Cluster	Marker	log2(fold change)	Adjusted p value	π-score
C6	CD142	0.40	5.6e-190	74.80
C6	CD163	0.42	1.9e-176	74.27
C6	CD116	0.41	1.2e-181	73.67
C6	CD134 (OX40)	0.44	8.0e-166	72.53
C6	CD45RA	0.37	1.4e-186	69.07
C6	CD137 (4-1BB)	0.39	5.6e-176	67.86
C6	CD158e1 (KIR3DL1, NKB1)	0.34	5.4e-195	66.70
C6	Podoplanin	0.42	2.8e-159	66.24
C6	CD109	0.39	2.2e-166	63.91
C6	CD314 (NKG2D)	0.41	1.5e-145	59.78
C6	CD112 (Nectin-2)	0.36	1.3e-151	53.65
C6	CD154	0.38	2.1e-133	50.26
C6	CD93	0.32	4.3e-155	49.73
C6	GPR56	0.32	4.7e-153	48.54
C6	CD14	0.29	1.9e-161	46.26
C6	Ig light chain λ	0.32	1.7e-138	44.23
C6	CD85j (ILT2)	0.34	5.3e-130	43.32
C6	CD150 (SLAM)	0.36	1.2e-119	42.36
C6	FcεRIα	0.30	9.8e-139	41.93
C6	CD354 (TREM-1)	0.27	1.3e-146	39.18
C6	CD158 (KIR2DL1/S1/S3/S5)	0.26	5.4e-147	38.68
C6	CD13	0.28	4.6e-135	37.48
C6	KLRG1 (MAFA)	0.31	1.1e-118	36.35
C6	CD195 (CCR5)	0.31	6.5e-113	35.14
C6	Ig light chain κ	0.29	1.0e-117	34.49
C6	CD196 (CCR6)	0.32	2.0e-107	34.34
C6	CD319 (CRACC)	0.27	6.6e-117	31.72
C6	CD152 (CTLA-4)	0.26	5.4e-118	30.95
C6	CD122 (IL-2Rβ)	0.30	3.3e-101	29.85
C7	CD49f	0.48	9.8e-213	101.02
C7	CD127 (IL-7Rα)	0.39	3.0e-183	71.50
C7	CD26	0.29	5.8e-108	30.76
C8	CD314 (NKG2D)	0.90	1.1e-161	144.42
C8	CD244 (2B4)	0.58	3.6e-114	65.60
C8	CD8	0.59	1.6e-110	64.44
C8	CD195 (CCR5)	0.47	2.9e-82	38.15
C8	CD122 (IL-2Rβ)	0.41	1.1e-87	35.73
C8	KLRG1 (MAFA)	0.39	1.1e-55	21.51
C8	CX3CR1	0.45	3.1e-31	13.79
C8	CD38	0.26	6.1e-40	10.39
C8	GPR56	0.29	6.2e-35	9.94
C8	CD24	0.36	1.8e-27	9.54
C8	CD223 (LAG-3)	0.36	6.9e-27	9.46
C8	LOX-1	0.34	7.0e-25	8.26
C8	CD79b (Igβ)	0.34	4.7e-24	7.85
C8	CD74	0.36	3.0e-22	7.82
C8	IgE	0.31	7.8e-26	7.68
C8	CD303 (BDCA-2)	0.33	1.2e-21	6.82
C8	CD23	0.34	3.2e-18	5.96
C8	CD163	0.26	3.8e-17	4.22
C8	CD83	0.27	2.0e-15	4.03
C8	CD134 (OX40)	0.25	2.5e-09	2.16
C9	CD1c	1.14	1.7e-185	210.39
C9	CD116	1.27	2.2e-159	200.73
C9	CD86	1.04	7.0e-183	188.71
C9	HLA-DR	1.03	2.7e-148	151.94

(continued)

Cluster	Marker	log2(fold change)	Adjusted p value	π-score
C9	CD40	1.01	1.3e-137	137.73
C9	HLA-DR, DP, DQ	1.00	7.2e-130	129.55
C9	CD131	0.84	8.8e-151	126.05
C9	CD39	0.98	4.6e-128	125.09
C9	CD54	0.93	1.7e-132	121.89
C9	CD112 (Nectin-2)	0.96	6.8e-122	116.20
C9	CD274 (B7-H1, PD-L1)	0.95	1.1e-122	116.03
C9	CD58 (LFA-3)	0.72	5.2e-112	80.62
C9	CD141 (Thrombomodulin)	0.85	1.6e-89	75.49
C9	CD32	0.87	1.5e-85	73.78
C9	CD9	0.68	1.6e-80	54.39
C9	CD83	0.55	7.1e-72	39.01
C9	CD63	0.55	1.7e-68	37.55
C9	CD119 (IFN-γ R α chain)	0.52	5.1e-72	36.72
C9	CD155 (PVR)	0.43	5.1e-53	22.56
C9	CD304 (Neuropilin-1)	0.40	7.7e-57	22.40
C9	CD31	0.40	1.4e-52	21.00
C9	CD85j (ILT2)	0.40	1.2e-52	20.84
C9	CD319 (CRACC)	0.37	1.3e-54	20.17
C9	CD72	0.41	1.3e-48	19.79
C9	CD267 (TACI)	0.32	7.1e-57	18.08
C9	CD13	0.26	1.1e-32	8.18
C9	CD169 (Sialoadhesin, Siglec-1)	0.25	6.9e-28	6.90
C9	IgE	0.26	3.4e-24	6.10
C9	CD49d	0.26	4.2e-23	5.72

Supplemental Table 6: Significant surface markers enriched in HIV+ versus HIV- cells during chronic infection. Test performed using the DESeq2 pseudobulk method in Seurat (two-tailed with multiple comparison adjustment using Bonferroni-Hochberg method).

Subset	Cluster	Marker	log2(fold change)	Adjusted p value	π-score
CD4+	HIV-	CD48	-0.58	1.2e-04	-2.25
CD4+	HIV-	CD49f	-0.91	3.0e-02	-1.39
CD4+	HIV-	CD47	-0.56	2.8e-02	-0.87
CD4+	HIV+	CD71	1.98	9.5e-15	27.72
CD4+	HIV+	CD278 (ICOS)	1.16	7.0e-06	5.98
CD4+	HIV+	HLA-DR	0.91	6.8e-04	2.89
CD4+	HIV+	HLA-DR, DP, DQ	1.04	5.0e-03	2.39
CD4+	HIV+	CD279 (PD-1)	1.17	3.5e-02	1.70
CD4+	HIV+	CD151 (PETA-3)	0.76	3.3e-02	1.12
Tfh CD4+	HIV-	CD69	-0.79	1.9e-02	-1.36
Tfh CD4+	HIV-	CD45	-0.31	1.6e-03	-0.87
Tfh CD4+	HIV+	CD71	1.19	3.9e-08	8.79

Supplemental Table 7: Significant surface markers enriched in each cluster (over all other cells) during treated infection. Significance calculated in Seurat using two-sided Wilcoxon test with multiple comparison adjustment using Bonferroni-Hochberg method.

Cluster	Marker	log2(fold change)	Adjusted p value	π-score
C1	CD71	1.70	4.4e-262	445.49
C1	CD31	1.23	4.2e-251	306.72
C1	CD38	1.11	3.8e-234	259.30
C1	CD112 (Nectin-2)	0.99	4.8e-248	244.33
C1	HLA-DR	1.03	1.0e-190	195.96
C1	CD244 (2B4)	0.90	4.5e-204	182.49
C1	HLA-DR, DP, DQ	0.94	9.1e-193	179.78
C1	CD123	0.62	8.8e-267	165.89
C1	CD33	0.71	8.0e-192	135.51
C1	CD49b	0.67	4.9e-157	105.12
C1	CD305 (LAIR1)	0.58	9.3e-138	79.08
C1	CD200 (OX2)	0.59	9.2e-124	72.53
C1	CD109	0.44	2.9e-81	35.20
C1	CLEC12A	0.43	7.3e-71	29.85
C1	GPR56	0.47	8.9e-62	28.43
C1	CD151 (PETA-3)	0.36	1.7e-74	26.86
C1	CD63	0.35	1.3e-70	24.36
C1	CD131	0.36	1.5e-51	18.11
C1	CD9	0.25	1.2e-39	9.74
C10	GPR56	0.49	0.0e+00	Inf
C10	CD57	0.41	0.0e+00	Inf
C10	CD244 (2B4)	0.34	0.0e+00	Inf
C10	KLRG1 (MAFA)	0.32	0.0e+00	Inf
C11	CD94	1.21	0.0e+00	Inf
C11	CD56 (NCAM)	1.20	0.0e+00	Inf
C11	CD16	0.87	0.0e+00	Inf
C11	CD314 (NKG2D)	0.85	0.0e+00	Inf
C11	CD244 (2B4)	0.85	0.0e+00	Inf
C11	CD11c	0.79	0.0e+00	Inf
C11	GPR56	0.74	0.0e+00	Inf
C11	CD9	0.64	0.0e+00	Inf
C11	NKp80	0.61	0.0e+00	Inf
C11	CD63	0.59	0.0e+00	Inf
C11	CD57	0.79	1.6e-264	207.30
C11	CD61	0.61	2.8e-288	176.60
C11	CD41	0.64	8.7e-262	166.86
C11	CD31	0.58	6.7e-269	154.27
C11	CD8	0.61	5.2e-222	134.10
C11	CD151 (PETA-3)	0.42	7.3e-231	96.01
C11	CLEC1B (CLEC2)	0.47	3.2e-194	91.55
C11	CD122 (IL-2Rβ)	0.39	2.5e-217	84.64
C11	CD328 (Siglec-7)	0.48	1.1e-169	81.74
C11	CD11b	0.29	2.2e-243	70.62
C11	CD32	0.28	6.6e-208	57.34
C11	CD38	0.39	1.9e-120	46.65
C11	TIGIT (VSTM3)	0.34	9.6e-137	46.63
C11	HLA-DR	0.29	3.6e-119	34.06
C11	CD49b	0.34	4.0e-55	18.72
C11	CD158e1 (KIR3DL1, NKB1)	0.41	3.3e-03	1.02
C12	GPR56	0.86	0.0e+00	Inf
C12	CD57	0.86	0.0e+00	Inf
C12	KLRG1 (MAFA)	0.76	0.0e+00	Inf
C12	CX3CR1	0.54	0.0e+00	Inf
C12	CD74	0.45	0.0e+00	Inf

(continued)

Cluster	Marker	log2(fold change)	Adjusted p value	π-score
C12	CD122 (IL-2Rβ)	0.38	0.0e+00	Inf
C12	CD244 (2B4)	0.36	0.0e+00	Inf
C12	CD79b (Igβ)	0.35	0.0e+00	Inf
C12	CD23	0.26	0.0e+00	Inf
C13	GPR56	0.87	0.0e+00	Inf
C13	KLRG1 (MAFA)	0.73	0.0e+00	Inf
C13	CD57	0.54	0.0e+00	Inf
C13	CD244 (2B4)	0.50	0.0e+00	Inf
C13	CD319 (CRACC)	0.32	0.0e+00	Inf
C13	CD195 (CCR5)	0.27	0.0e+00	Inf
C15	CD69	0.48	0.0e+00	Inf
C15	CD49f	0.28	0.0e+00	Inf
C16	CD74	0.49	0.0e+00	Inf
C16	CD79b (Igβ)	0.38	0.0e+00	Inf
C16	CD55	0.34	0.0e+00	Inf
C16	CD62L	0.31	0.0e+00	Inf
C16	CD23	0.30	0.0e+00	Inf
C16	CD200 (OX2)	0.30	0.0e+00	Inf
C17	CD49b	0.34	0.0e+00	Inf
C17	CD9	0.32	0.0e+00	Inf
C17	CD62L	0.27	0.0e+00	Inf
C17	CD61	0.26	0.0e+00	Inf
C17	CD39	0.26	0.0e+00	Inf
C18	CD161	0.54	0.0e+00	Inf
C18	CD26	0.53	0.0e+00	Inf
C18	CD55	0.46	0.0e+00	Inf
C18	CD192 (CCR2)	0.44	0.0e+00	Inf
C18	CD49f	0.43	0.0e+00	Inf
C18	CD73 (Ecto-5'-nucleotidase)	0.42	0.0e+00	Inf
C18	CD127 (IL-7Rα)	0.39	0.0e+00	Inf
C18	CD27	0.37	0.0e+00	Inf
C18	CD7	0.36	0.0e+00	Inf
C18	CD150 (SLAM)	0.32	0.0e+00	Inf
C18	CD49d	0.28	0.0e+00	Inf
C18	CD69	0.28	0.0e+00	Inf
C18	CD196 (CCR6)	0.26	0.0e+00	Inf
C18	CD29	0.26	0.0e+00	Inf
C18	CD35	0.29	1.1e-209	60.22
C19	CD25	0.71	0.0e+00	Inf
C19	CD39	0.61	0.0e+00	Inf
C19	TIGIT (VSTM3)	0.55	0.0e+00	Inf
C19	CD71	0.53	0.0e+00	Inf
C19	HLA-DR, DP, DQ	0.48	0.0e+00	Inf
C19	HLA-DR	0.45	0.0e+00	Inf
C19	CD194 (CCR4)	0.43	0.0e+00	Inf
C19	CD101 (BB27)	0.40	0.0e+00	Inf
C19	CD27	0.34	0.0e+00	Inf
C19	CD278 (ICOS)	0.28	0.0e+00	Inf
C19	CD95 (Fas)	0.28	0.0e+00	Inf
C2	CD11c	1.13	0.0e+00	Inf
C2	CD141 (Thrombomodulin)	0.84	0.0e+00	Inf
C2	CLEC12A	0.80	0.0e+00	Inf
C2	CD116	0.76	0.0e+00	Inf
C2	CD328 (Siglec-7)	0.55	0.0e+00	Inf
C2	CD33	0.90	1.5e-248	222.74
C2	CD93	0.72	1.2e-305	218.20

(continued)

Cluster	Marker	log2(fold change)	Adjusted p value	π-score
C2	HLA-DR	0.88	1.8e-178	157.02
C2	CD32	0.58	2.8e-247	142.85
C2	CD13	0.45	3.0e-266	120.17
C2	CD31	0.67	6.4e-153	102.63
C2	CD38	0.45	9.6e-200	89.01
C2	CD85j (ILT2)	0.41	2.1e-203	82.12
C2	FcεRIα	0.56	2.1e-143	79.49
C2	CD63	0.49	4.2e-159	77.35
C2	HLA-DR, DP, DQ	0.80	2.1e-95	75.54
C2	CD39	0.47	1.1e-155	72.74
C2	CD35	0.48	5.6e-128	61.31
C2	IgE	0.47	3.9e-128	60.26
C2	CD354 (TREM-1)	0.31	3.4e-193	59.58
C2	CD49b	0.44	1.2e-133	58.84
C2	CD112 (Nectin-2)	0.41	4.3e-143	58.62
C2	CD14	0.37	3.2e-131	47.66
C2	CD9	0.52	3.7e-92	47.10
C2	CD61	0.52	3.9e-84	43.64
C2	CD41	0.53	2.1e-79	42.00
C2	CD88 (C5aR)	0.26	5.4e-158	41.45
C2	CD74	0.45	5.5e-88	39.11
C2	CLEC1B (CLEC2)	0.39	1.6e-89	34.26
C2	CD163	0.35	1.6e-91	32.22
C2	CD83	0.34	2.1e-84	28.69
C2	CD62P (P-Selectin)	0.32	5.3e-89	28.19
C2	CD101 (BB27)	0.31	9.9e-84	26.11
C2	CD119 (IFN-γ R α chain)	0.26	1.3e-93	24.46
C2	CD200 (OX2)	0.32	7.8e-70	21.80
C2	LOX-1	0.29	4.2e-40	11.38
C2	CD23	0.31	1.9e-29	9.03
C2	CD54	0.30	1.3e-26	7.66
C2	CD79b (Igβ)	0.30	3.3e-16	4.63
C20	CD27	0.49	0.0e+00	Inf
C20	CD7	0.48	0.0e+00	Inf
C20	integrin β7	0.32	0.0e+00	Inf
C21	CD74	0.52	0.0e+00	Inf
C21	CD79b (Igβ)	0.42	0.0e+00	Inf
C21	CD23	0.35	0.0e+00	Inf
C21	CD200 (OX2)	0.34	0.0e+00	Inf
C21	CD163	0.25	0.0e+00	Inf
C21	LOX-1	0.25	3.6e-289	73.25
C3	CD35	1.17	0.0e+00	Inf
C3	CD32	1.10	0.0e+00	Inf
C3	HLA-DR, DP, DQ	1.07	0.0e+00	Inf
C3	HLA-DR	1.06	0.0e+00	Inf
C3	CD31	0.72	0.0e+00	Inf
C3	CD40	0.61	0.0e+00	Inf
C3	IgM	0.76	3.7e-191	145.03
C3	CD39	0.58	1.3e-240	139.96
C3	CD9	0.57	4.6e-199	112.67
C3	CD37	0.44	3.7e-242	106.96
C3	CD71	0.58	1.1e-183	105.31
C3	CD185 (CXCR5)	0.46	3.8e-208	95.11
C3	CD41	0.59	1.1e-158	92.74
C3	CD61	0.54	7.8e-143	76.61
C3	CD38	0.42	2.0e-146	61.55

(continued)

Cluster	Marker	log2(fold change)	Adjusted p value	π-score
C3	CD73 (Ecto-5’-nucleotidase)	0.50	2.6e-119	59.52
C3	CD200 (OX2)	0.39	2.3e-119	45.97
C3	Ig light chain λ	0.51	4.3e-87	43.69
C3	CD63	0.33	4.9e-124	40.74
C3	CLEC1B (CLEC2)	0.37	7.4e-104	38.03
C3	CD275 (B7-H2, B7-RP1, ICOSL)	0.32	9.7e-119	37.52
C3	CD85j (ILT2)	0.30	5.3e-122	36.69
C3	Ig light chain κ	0.37	7.7e-94	34.66
C3	CD24	0.28	8.5e-102	28.25
C3	CD305 (LAIR1)	0.32	3.3e-85	26.66
C3	CD49b	0.39	2.9e-67	25.95
C3	CD74	0.27	1.2e-72	19.15
C3	CD11c	0.27	5.3e-61	16.15
C3	CD272 (BTLA)	0.27	4.2e-57	15.11
C3	CD42b	0.26	2.9e-36	9.29
C4	CD11c	1.33	0.0e+00	Inf
C4	CD33	1.24	0.0e+00	Inf
C4	CLEC12A	1.12	0.0e+00	Inf
C4	CD35	1.03	0.0e+00	Inf
C4	CD31	1.00	0.0e+00	Inf
C4	HLA-DR	0.97	0.0e+00	Inf
C4	CD328 (Siglec-7)	0.77	0.0e+00	Inf
C4	CD116	0.75	0.0e+00	Inf
C4	HLA-DR, DP, DQ	0.72	0.0e+00	Inf
C4	CD63	0.70	0.0e+00	Inf
C4	CD32	0.64	0.0e+00	Inf
C4	CD9	0.63	0.0e+00	Inf
C4	CD141 (Thrombomodulin)	0.62	0.0e+00	Inf
C4	CD11b	0.62	0.0e+00	Inf
C4	CD41	0.62	0.0e+00	Inf
C4	CD61	0.60	0.0e+00	Inf
C4	CD88 (C5aR)	0.58	0.0e+00	Inf
C4	CD93	0.54	0.0e+00	Inf
C4	CD38	0.43	0.0e+00	Inf
C4	CD354 (TREM-1)	0.43	0.0e+00	Inf
C4	CD39	0.42	0.0e+00	Inf
C4	CD85j (ILT2)	0.41	0.0e+00	Inf
C4	CD244 (2B4)	0.37	0.0e+00	Inf
C4	CD13	0.36	0.0e+00	Inf
C4	CD192 (CCR2)	0.40	1.4e-281	112.97
C4	CD14	0.42	3.6e-271	112.45
C4	CLEC1B (CLEC2)	0.39	1.6e-255	99.63
C4	CD101 (BB27)	0.32	1.9e-263	83.01
C4	CD62P (P-Selectin)	0.31	2.5e-164	50.18
C4	CD49b	0.33	1.9e-149	48.88
C4	FcεRIα	0.26	2.8e-48	12.22
C6	CD74	0.86	0.0e+00	Inf
C6	CD79b (Igβ)	0.82	0.0e+00	Inf
C6	IgE	0.76	0.0e+00	Inf
C6	CD23	0.76	0.0e+00	Inf
C6	CD134 (OX40)	0.70	0.0e+00	Inf
C6	LOX-1	0.69	0.0e+00	Inf
C6	CD314 (NKG2D)	0.66	0.0e+00	Inf
C6	CD200 (OX2)	0.64	0.0e+00	Inf
C6	CD24	0.63	0.0e+00	Inf
C6	CD83	0.62	0.0e+00	Inf

(continued)

Cluster	Marker	log2(fold change)	Adjusted p value	π-score
C6	CD163	0.62	0.0e+00	Inf
C6	CD45RA	0.60	0.0e+00	Inf
C6	CX3CR1	0.57	0.0e+00	Inf
C6	CD93	0.55	0.0e+00	Inf
C6	CD141 (Thrombomodulin)	0.53	0.0e+00	Inf
C6	CD303 (BDCA-2)	0.52	0.0e+00	Inf
C6	CD116	0.50	0.0e+00	Inf
C6	CD112 (Nectin-2)	0.50	0.0e+00	Inf
C6	CD275 (B7-H2, B7-RP1, ICOSL)	0.48	0.0e+00	Inf
C6	CD13	0.46	0.0e+00	Inf
C6	CD122 (IL-2Rβ)	0.45	0.0e+00	Inf
C6	CD183 (CXCR3)	0.44	0.0e+00	Inf
C6	CD196 (CCR6)	0.43	0.0e+00	Inf
C6	FcεRIα	0.42	0.0e+00	Inf
C6	CD115 (CSF-1R)	0.42	0.0e+00	Inf
C6	CD223 (LAG-3)	0.41	0.0e+00	Inf
C6	Podoplanin	0.40	0.0e+00	Inf
C6	CD142	0.39	0.0e+00	Inf
C6	NKp80	0.38	0.0e+00	Inf
C6	CD158 (KIR2DL1/S1/S3/S5)	0.38	0.0e+00	Inf
C6	CD14	0.37	0.0e+00	Inf
C6	CD85j (ILT2)	0.37	0.0e+00	Inf
C6	CD109	0.36	0.0e+00	Inf
C6	CD169 (Sialoadhesin, Siglec-1)	0.35	0.0e+00	Inf
C6	CD195 (CCR5)	0.33	0.0e+00	Inf
C6	CD152 (CTLA-4)	0.31	0.0e+00	Inf
C6	CD185 (CXCR5)	0.31	0.0e+00	Inf
C6	CD354 (TREM-1)	0.30	0.0e+00	Inf
C6	CD158e1 (KIR3DL1, NKB1)	0.29	0.0e+00	Inf
C6	CD94	0.28	0.0e+00	Inf
C6	CD137 (4-1BB)	0.28	0.0e+00	Inf
C6	CD279 (PD-1)	0.28	0.0e+00	Inf
C6	TIGIT (VSTM3)	0.27	0.0e+00	Inf
C6	CD38	0.26	0.0e+00	Inf
C6	CD107a (LAMP-1)	0.26	0.0e+00	Inf
C7	CD83	0.59	0.0e+00	Inf
C7	CD303 (BDCA-2)	0.56	0.0e+00	Inf
C7	CD223 (LAG-3)	0.54	0.0e+00	Inf
C7	CD275 (B7-H2, B7-RP1, ICOSL)	0.53	0.0e+00	Inf
C7	CD24	0.52	0.0e+00	Inf
C7	NKp80	0.50	0.0e+00	Inf
C7	CD169 (Sialoadhesin, Siglec-1)	0.49	0.0e+00	Inf
C7	CD134 (OX40)	0.49	0.0e+00	Inf
C7	IgE	0.47	0.0e+00	Inf
C7	CD23	0.46	0.0e+00	Inf
C7	CD137 (4-1BB)	0.46	0.0e+00	Inf
C7	LOX-1	0.44	0.0e+00	Inf
C7	CD116	0.43	0.0e+00	Inf
C7	Podoplanin	0.43	0.0e+00	Inf
C7	CD142	0.42	0.0e+00	Inf
C7	CD109	0.42	0.0e+00	Inf
C7	CD112 (Nectin-2)	0.42	0.0e+00	Inf
C7	CD163	0.39	0.0e+00	Inf
C7	CD115 (CSF-1R)	0.38	0.0e+00	Inf
C7	CD85j (ILT2)	0.38	0.0e+00	Inf
C7	CD74	0.37	0.0e+00	Inf

(continued)

Cluster	Marker	log2(fold change)	Adjusted p value	π-score
C7	CD152 (CTLA-4)	0.36	0.0e+00	Inf
C7	CD79b (Igβ)	0.35	0.0e+00	Inf
C7	CD200 (OX2)	0.32	0.0e+00	Inf
C7	CD304 (Neuropilin-1)	0.32	0.0e+00	Inf
C7	FcεRIα	0.31	0.0e+00	Inf
C7	Ig light chain κ	0.31	0.0e+00	Inf
C7	CD314 (NKG2D)	0.31	0.0e+00	Inf
C7	CD45RA	0.30	0.0e+00	Inf
C7	CX3CR1	0.29	0.0e+00	Inf
C7	CD141 (Thrombomodulin)	0.29	0.0e+00	Inf
C7	CD158e1 (KIR3DL1, NKB1)	0.29	0.0e+00	Inf
C7	CD14	0.28	0.0e+00	Inf
C7	TIGIT (VSTM3)	0.28	0.0e+00	Inf
C7	CD93	0.27	0.0e+00	Inf
C7	CD267 (TACI)	0.27	0.0e+00	Inf
C7	CD37	0.26	0.0e+00	Inf
C7	CD158 (KIR2DL1/S1/S3/S5)	0.25	0.0e+00	Inf
C8	CD116	0.52	1.6e-217	112.70
C8	CX3CR1	0.54	3.3e-203	110.10
C8	CD79b (Igβ)	0.53	4.6e-207	110.06
C8	CD83	0.50	7.5e-178	88.54
C8	CD303 (BDCA-2)	0.44	2.5e-165	73.24
C8	CD134 (OX40)	0.42	4.1e-172	72.71
C8	IgM	0.38	1.5e-141	52.91
C8	CD23	0.35	1.3e-140	48.77
C8	IgE	0.33	6.7e-136	45.07
C8	CD74	0.34	6.9e-132	44.67
C8	CD183 (CXCR3)	0.33	6.1e-132	43.07
C8	CD194 (CCR4)	0.34	1.0e-126	42.33
C8	CD24	0.32	7.0e-119	37.70
C8	Ig light chain λ	0.30	2.7e-123	37.32
C8	CD141 (Thrombomodulin)	0.29	1.5e-126	36.31
C8	LOX-1	0.29	7.2e-114	33.31
C8	CD112 (Nectin-2)	0.28	5.4e-105	29.68
C8	CD200 (OX2)	0.29	2.4e-102	29.08
C8	CD142	0.25	1.3e-95	24.08
C8	CD195 (CCR5)	0.28	1.6e-86	23.88
C9	CD195 (CCR5)	0.83	1.6e-167	138.19
C9	CD161	0.83	1.7e-129	107.44
C9	CD94	0.83	7.1e-63	51.79
C9	KLRG1 (MAFA)	0.58	2.5e-82	47.27
C9	CD69	0.53	2.8e-66	34.69
C9	CD26	0.41	1.6e-76	30.70
C9	CD314 (NKG2D)	0.45	1.9e-68	30.41
C9	CD56 (NCAM)	0.45	1.0e-48	21.46
C9	CD119 (IFN-γ R α chain)	0.31	4.0e-58	18.06
C9	CD8	0.48	1.1e-31	14.72
C9	CD244 (2B4)	0.32	5.6e-41	12.79
C9	CD31	0.30	3.5e-29	8.49
C9	integrin β7	0.25	7.5e-31	7.59
C9	CD7	0.25	7.4e-26	6.30
C9	CD41	0.26	5.3e-19	4.84

Supplemental Table 8: Significant surface markers enriched in HIV+ versus HIV- cells during treated infection. Test performed using the DESeq2 pseudobulk method in Seurat (two-tailed with multiple comparison adjustment using Bonferroni-Hochberg method).

Subset	Cluster	Marker	log2(fold change)	Adjusted p value	π-score
CD4+ (DESeq2)	HIV-	CD74	-0.86	7.6e-15	-12.14
CD4+ (DESeq2)	HIV-	CD41	-0.80	8.0e-11	-8.07
CD4+ (DESeq2)	HIV-	CD314 (NKG2D)	-0.61	2.2e-08	-4.67
CD4+ (DESeq2)	HIV-	CD158e1 (KIR3DL1, NKB1)	-0.69	3.9e-07	-4.44
CD4+ (DESeq2)	HIV-	CD61	-0.60	1.3e-07	-4.13
CD4+ (DESeq2)	HIV-	CD8	-0.61	2.2e-07	-4.06
CD4+ (DESeq2)	HIV-	GPR56	-0.67	1.5e-06	-3.90
CD4+ (DESeq2)	HIV-	LOX-1	-0.50	3.8e-07	-3.19
CD4+ (DESeq2)	HIV-	CD305 (LAIR1)	-0.32	1.5e-09	-2.84
CD4+ (DESeq2)	HIV-	CD122 (IL-2Rβ)	-0.36	4.0e-07	-2.30
CD4+ (DESeq2)	HIV-	CD49b	-0.49	3.3e-05	-2.21
CD4+ (DESeq2)	HIV-	CD56 (NCAM)	-0.46	4.6e-05	-2.01
CD4+ (DESeq2)	HIV-	CLEC1B (CLEC2)	-0.44	6.2e-05	-1.84
CD4+ (DESeq2)	HIV-	CD163	-0.46	1.0e-04	-1.83
CD4+ (DESeq2)	HIV-	CD79b (Igβ)	-0.53	3.6e-04	-1.82
CD4+ (DESeq2)	HIV-	CD101 (BB27)	-0.39	5.1e-05	-1.69
CD4+ (DESeq2)	HIV-	CD3	-0.16	2.0e-11	-1.69
CD4+ (DESeq2)	HIV-	CD134 (OX40)	-0.43	4.6e-04	-1.45
CD4+ (DESeq2)	HIV-	CD94	-0.45	1.1e-03	-1.34
CD4+ (DESeq2)	HIV-	CD31	-0.40	5.0e-04	-1.33
CD4+ (DESeq2)	HIV-	CD45RA	-0.41	2.3e-03	-1.07
CD4+ (DESeq2)	HIV-	CD23	-0.44	7.1e-03	-0.95
CD4+ (DESeq2)	HIV-	CD141 (Thrombomodulin)	-0.36	4.5e-03	-0.85
CD4+ (DESeq2)	HIV-	CD151 (PETA-3)	-0.22	1.8e-04	-0.83
CD4+ (DESeq2)	HIV-	CD62L	-0.12	2.4e-07	-0.82
CD4+ (DESeq2)	HIV-	CD13	-0.31	4.3e-03	-0.74
CD4+ (DESeq2)	HIV-	CD35	-0.42	4.6e-02	-0.57
CD4+ (DESeq2)	HIV-	CD244 (2B4)	-0.21	6.9e-03	-0.45
CD4+ (DESeq2)	HIV-	CD14	-0.27	2.5e-02	-0.43
CD4+ (DESeq2)	HIV-	CD62P (P-Selectin)	-0.12	1.4e-02	-0.23
CD4+ (DESeq2)	HIV-	CD82	-0.04	2.9e-02	-0.06
CD4+ (DESeq2)	HIV-	TCR α/β	-0.01	2.6e-02	-0.02
CD4+ (DESeq2)	HIV+	CD195 (CCR5)	0.63	1.0e-04	2.51
CD4+ (DESeq2)	HIV+	CD279 (PD-1)	0.47	7.5e-05	1.94
CD4+ (DESeq2)	HIV+	CD2	0.33	1.5e-03	0.94
CD4+ (Wilcoxon)	HIV-	CD305 (LAIR1)	-0.15	8.6e-05	-0.60
CD4+ (Wilcoxon)	HIV+	CD150 (SLAM)	0.17	1.9e-09	1.51
CD4+ (Wilcoxon)	HIV+	CD195 (CCR5)	0.23	1.0e-05	1.13
CD4+ (Wilcoxon)	HIV+	CD279 (PD-1)	0.17	4.3e-07	1.07
CD4+ (Wilcoxon)	HIV+	CD49d	0.10	8.8e-04	0.32
CD4+ (Wilcoxon)	HIV+	HLA-DR	0.11	2.6e-03	0.30
CD4+ (Wilcoxon)	HIV+	CD192 (CCR2)	0.13	6.3e-03	0.28
CD4+ (Wilcoxon)	HIV+	HLA-DR, DP, DQ	0.14	3.7e-02	0.20
Tcm/Ttm (DESeq2)	HIV-	CD103 (Integrin αE)	-1.04	3.6e-03	-2.55
Tcm/Ttm (DESeq2)	HIV-	CD74	-0.60	1.5e-02	-1.09
Tcm/Ttm (DESeq2)	HIV-	CD62L	-0.23	5.4e-04	-0.75
Tcm/Ttm (DESeq2)	HIV-	CD55	-0.24	2.4e-02	-0.40
Tcm/Ttm (DESeq2)	HIV+	CD57	1.53	6.8e-11	15.55
Tcm/Ttm (DESeq2)	HIV+	CD195 (CCR5)	1.03	3.0e-07	6.71
Tcm/Ttm (DESeq2)	HIV+	CD2	0.53	2.6e-11	5.64
Tcm/Ttm (DESeq2)	HIV+	KLRG1 (MAFA)	0.96	2.7e-04	3.41
Tcm/Ttm (DESeq2)	HIV+	CD11a	0.47	3.0e-06	2.60
Tcm/Ttm (DESeq2)	HIV+	CD279 (PD-1)	0.61	8.4e-04	1.87
Tcm/Ttm (DESeq2)	HIV+	HLA-DR, DP, DQ	0.70	1.3e-02	1.32

(continued)

Subset	Cluster	Marker	log2(fold change)	Adjusted p value	π-score
Tcm/Ttm (DESeq2)	HIV+	CD18	0.42	2.4e-02	0.69
Tcm/Ttm (DESeq2)	HIV+	CD26	0.46	3.2e-02	0.68
Tcm/Ttm (DESeq2)	HIV+	CD71	0.49	4.4e-02	0.67
Tem (DESeq2)	HIV-	CD41	-1.47	5.6e-07	-9.21
Tem (DESeq2)	HIV-	CD305 (LAIR1)	-0.78	1.4e-07	-5.36
Tem (DESeq2)	HIV-	CD101 (BB27)	-1.10	3.1e-05	-4.97
Tem (DESeq2)	HIV-	CD61	-1.13	7.8e-05	-4.65
Tem (DESeq2)	HIV-	CD3	-0.50	2.9e-08	-3.78
Tem (DESeq2)	HIV-	GPR56	-0.86	4.1e-05	-3.76
Tem (DESeq2)	HIV-	CD56 (NCAM)	-0.98	2.2e-03	-2.60
Tem (DESeq2)	HIV-	CD71	-0.90	2.1e-03	-2.41
Tem (DESeq2)	HIV-	CD122 (IL-2Rβ)	-0.73	6.2e-04	-2.36
Tem (DESeq2)	HIV-	CD200 (OX2)	-1.08	7.9e-03	-2.27
Tem (DESeq2)	HIV-	CD8	-1.04	8.6e-03	-2.15
Tem (DESeq2)	HIV-	CD158e1 (KIR3DL1, NKB1)	-1.21	2.0e-02	-2.06
Tem (DESeq2)	HIV-	CD38	-0.78	6.0e-03	-1.74
Tem (DESeq2)	HIV-	CLEC1B (CLEC2)	-0.81	1.0e-02	-1.62
Tem (DESeq2)	HIV-	CD151 (PETA-3)	-0.56	1.3e-03	-1.61
Tem (DESeq2)	HIV-	LOX-1	-0.84	2.3e-02	-1.37
Tem (DESeq2)	HIV-	CD314 (NKG2D)	-0.88	2.9e-02	-1.36
Tem (DESeq2)	HIV-	CD93	-0.61	2.6e-02	-0.97
Tem (DESeq2)	HIV-	CD5	-0.27	7.7e-04	-0.83
Tem (DESeq2)	HIV-	CD244 (2B4)	-0.34	3.4e-02	-0.50
Tem (DESeq2)	HIV+	CD279 (PD-1)	0.58	7.1e-06	2.98
Tem (DESeq2)	HIV+	CD195 (CCR5)	0.59	2.1e-02	0.99

Supplemental Table 9: Autologous or consensus virus sequences used in study.

Individual	Fasta Prefix or Name	Location	Publication
A08	chrA08	GenBank; Supplementary File 1	Gondim et al., 2021
A09	chrA09	GenBank; Supplementary File 1	Gondim et al., 2021
A01	chrA01.	Supplementary File 1	This paper
B45	chrBEAT2045_	Supplementary File 1	This paper
C01	HXB2	GenBank (K03455.1)	Ratner et al., 1985
C02	HXB2	GenBank (K03455.1)	Ratner et al., 1985
ND492	SUMA_TF1	GenBank (JN944928.1)	Ochsenbauer et al., 2012
