# Supplementary material for: Profound phenotypic and epigenetic heterogeneity of the HIV-1-infected CD4+ T cell reservoir
Source: Nat Immunol. 2022 Dec 19;24(2):359–70. doi: 10.1038/s41590-022-01371-3 (PMC9892009; doi:10.1038/s41590-022-01371-3)
Supplement: Supplementary file 2 — Reporting Summary [file 41590_2022_1371_MOESM2_ESM.pdf]

nature portfolio

Corresponding author(s): Michael R. Betts and Laura A. Vella

Last updated by author(s): Sep 9, 2022

Reporting Summary

Nature Portfolio wishes to improve the reproducibility of the work that we publish. This form provides structure for consistency and transparency in reporting. For further information on Nature Portfolio policies, see our Editorial Policies and the Editorial Policy Checklist.

Statistics

For all statistical analyses, confirm that the following items are present in the figure legend, table legend, main text, or Methods section.

n/a	Confirmed	
☐	☒	The exact sample size (*n*) for each experimental group/condition, given as a discrete number and unit of measurement
☐	☒	A statement on whether measurements were taken from distinct samples or whether the same sample was measured repeatedly
☐	☒	The statistical test(s) used AND whether they are one- or two-sided<br>*Only common tests should be described solely by name; describe more complex techniques in the Methods section.*
☒	☐	A description of all covariates tested
☐	☒	A description of any assumptions or corrections, such as tests of normality and adjustment for multiple comparisons
☐	☒	A full description of the statistical parameters including central tendency (e.g. means) or other basic estimates (e.g. regression coefficient) AND variation (e.g. standard deviation) or associated estimates of uncertainty (e.g. confidence intervals)
☒	☐	For null hypothesis testing, the test statistic (e.g. *F*, *t*, *r*) with confidence intervals, effect sizes, degrees of freedom and *P* value noted<br>*Give P values as exact values whenever suitable.*
☒	☐	For Bayesian analysis, information on the choice of priors and Markov chain Monte Carlo settings
☒	☐	For hierarchical and complex designs, identification of the appropriate level for tests and full reporting of outcomes
☒	☐	Estimates of effect sizes (e.g. Cohen's *d*, Pearson's *r*), indicating how they were calculated

Our web collection on statistics for biologists contains articles on many of the points above.

Software and code

Policy information about availability of computer code

Data collection	cellranger-atac (v2.0.0); index-hopping-filter (v1.1); kallisto (v0.46.2); bustools (v0.40.0); AMULET (v1.1); ArchR (v1.0.2); Seurat (v4.1.1); chromVAR (v1.16.0); DropletUtils (v1.14.2); DESeq2 (v1.34.0); naivebayes (v0.9.7); caTools(v1.18.2), ROCR (v1.0-11); randomForest (v4.7-1.1); hiv-haystack (v1; https://github.com/betts-lab/hiv-haystack); Gene Cutter (HIV LANL Database; web version; https://www.hiv.lanl.gov/content/sequence/GENE_CUTTER/cutter.html); geneCutterParser (v1; https://github.com/wuv21/geneCutterParser); R (v4.1.1); gridExtra (v2.3); ggplot2 (v3.3.6); patchwork (v1.1.1)
Data analysis	Custom code is available at https://github.com/betts-lab/asapseq-hiv-art

For manuscripts utilizing custom algorithms or software that are central to the research but not yet described in published literature, software must be made available to editors and reviewers. We strongly encourage code deposition in a community repository (e.g. GitHub). See the Nature Portfolio guidelines for submitting code & software for further information.

Data

Policy information about availability of data

All manuscripts must include a data availability statement. This statement should provide the following information, where applicable:

- Accession codes, unique identifiers, or web links for publicly available datasets
- A description of any restrictions on data availability
- For clinical datasets or third party data, please ensure that the statement adheres to our policy

Raw fastq files and processed cellranger-atac files are deposited in the NCBI Gene Expression Omnibus (GEO) under series accession number GSE199727. CISBP human transcription factor motif data (http://cisbp.ccbr.utoronto.ca/) were included in the ArchR pacakge by the original developers. HIV LANL database (https://www.hiv.lanl.gov/content/sequence/GENE_CUTTER/cutter.html) was used to access the Gene Cutter tool.

Human research participants

Policy information about studies involving human research participants and Sex and Gender in Research.

Reporting on sex and gender	All untreated donors are male as reported by Centro de Investigación en Enfermedades Infecciosas. All treated donors are male as previously reported (Bar et al., 2016) and as reported for samples provided by the University of Pennsylvania Human Immunology Core and the BEAT-HIV program cohort.
Population characteristics	Age was not reported at the participant level for the original study (Bar et al., 2016). However, the age range was reported as between 34 to 52 years old. Donor ages for PLWH with chronic HIV infection are reported in Table 1. For the ex vivo data, all participants had diagnosis of HIV-1 infection and all had suppressed viral load under ART treatment at the time of sample collection. No genotypic information on participants was available for this study. Please refer to Table 1 for more information on all donors included in this study.
Recruitment	No participants were recruited specifically for this study. ART-treated samples were provided by Drs. Katie Bar, Pablo Tebas, and Luis Montaner from previously published studies. Chronic HIV samples were provided by Drs. del Rio Estrada, Torres-Ruiz, Gonzales-Navarro, Luna-Villalobos, Avila-Rios, and Reyes-Teran.
Ethics oversight	This study was approved by the Institutional Review Boards at the University of Pennsylvania and the University of Alabama at Birmingham. This study complies with all relevant ethical regulations. Persons living with chronic HIV (n = 2) were originally recruited by the Centro de Investigación en Enfermedades Infecciosas at the Instituto Nacional de Enfermedades Respiratorias (CIENI-INER) in Mexico City, Mexico. All donors provided informed consent for lymph node tissue donation in compliance with protocols set forth by the Ethics Committee and the Ethics in Research Committee of the INER (study number: B03-16) and the Institutional Review Board at the University of Pennsylvania (Philadelphia, PA). ART-treated samples (n = 3), A01, A08, and A09, were provided from the ACTG clinical trial A5340, which was conducted with protocols set forth by the Institutional Review Boards at the University of Pennsylvania and the University of Alabama at Birmingham and was previously published (Bar et al., 2016). The original clinical trial included provisions for research related to this study. All donors for this study provided informed consent in compliance with protocols set forth by the respective institutional review boards. Another ART-treated PLWH (n = 1), B45, was recruited from the BEAT-HIV program cohort where an apheresis sample was collected under ART. All participants were compensated for their time and study visits. No additional compensation was provided for this study.

Note that full information on the approval of the study protocol must also be provided in the manuscript.

Field-specific reporting

Please select the one below that is the best fit for your research. If you are not sure, read the appropriate sections before making your selection.

☒ Life sciences ☐ Behavioural & social sciences ☐ Ecological, evolutionary & environmental sciences

For a reference copy of the document with all sections, see nature.com/documents/nr-reporting-summary-flat.pdf

Life sciences study design

All studies must disclose on these points even when the disclosure is negative.

Sample size	2 separate ex vivo untreated samples (C01 and C02) were analyzed. 7 separate ex vivo treated samples were analyzed (A01 (two timepoints), A08 (two timepoints), A09 (two timepoints), and B45). No sample size calculations were performed as the samples were provided based on availability and rarity of infected cells.
Data exclusions	No datasets were excluded. Filtering of cells was done for each dataset for potential index hopping (if applicable), quality control, potential multiplets, and small (<0.5% of total number of QC passing cells) clusters due to the inability to annotate properly. A wilcoxon test (right-tailed) was used to select for surface antigen features with a count distribution that was significantly different than background signal

detected from isotype control antibodies, as analyzed similarly in a prior report (Swanson et al., 2021). A feature was not included in downstream analyses if p > 0.05 in at least 4 isotype controls.

Replication

No attempts at replication as these are direct ex vivo profiling of samples from individuals. In vitro experiment was not replicated as this was a proof of concept experiment and was confirmed for feasibility by the ex vivo experiments. Pseudo-bulk replicates were generated as part of the ArchR package in downstream differential analyses due to sparse single-cell epigenetic datasets (refer to ArchR documentation).

Randomization

This study was not designed as a clinical trial and all individuals were PLWH. The primary study measure was between cells within the same individual. Therefore, no randomization or blinding was performed in the study design or analysis.

Blinding

Similar to the above rationale, this study was not designed as a clinical trial and all individuals were PLWH. The primary study measure was between cells within the same individual. Therefore, no randomization or blinding was performed in the study design or analysis.

Reporting for specific materials, systems and methods

We require information from authors about some types of materials, experimental systems and methods used in many studies. Here, indicate whether each material, system or method listed is relevant to your study. If you are not sure if a list item applies to your research, read the appropriate section before selecting a response.

Materials & experimental systems

n/a	Involved in the study
☐	☒ Antibodies
☒	☐ Eukaryotic cell lines
☒	☐ Palaeontology and archaeology
☒	☐ Animals and other organisms
☒	☐ Clinical data
☒	☐ Dual use research of concern

Methods

n/a	Involved in the study
☒	☐ ChIP-seq
☐	☒ Flow cytometry
☒	☐ MRI-based neuroimaging

Antibodies

Antibodies used

All antibodies used are commercially available and are described in the Methods and in Supplemental Table 1.

For proteogenomics:
- TotalSeqA Human Universal Cocktail, V1.0 (BioLegend #399907)

For flow cytometry:
- CD8 BV570 (BioLegend #301038, clone RPA-T8)
- p24 FITC (Beckman Coulter #6604665; clone KC57)

Validation

All antibodies are published clones and validated by the vendors. For proteogenomic applications, the TotalSeqA Human Universal Cocktail (v1.0) was validated by BioLegend for proteogenomic applications and human reactivity. For flow cytometry, the CD8 BV570 antibody was validated by BioLegend for flow cytometry applications and human reactivity while the p24 FITC clone was validated by Beckman Coulter for flow cytometry applications and reactivity to HIV p24.

Flow Cytometry

Plots

Confirm that:

☒ The axis labels state the marker and fluorochrome used (e.g. CD4-FITC).

☒ The axis scales are clearly visible. Include numbers along axes only for bottom left plot of group (a 'group' is an analysis of identical markers).

☒ All plots are contour plots with outliers or pseudocolor plots.

☒ A numerical value for number of cells or percentage (with statistics) is provided.

Methodology

Sample preparation

Peripheral blood mononuclear cells (PBMCs) were obtained from an HIV-negative donor apheresis from the Human Immunology Core (HIC) at the University of Pennsylvania. Bulk CD4+ T cells were negatively enriched by bead separation and infected with HIV-1. Staining and flow cytometry were based on a previously published protocol (Kuri-Cervantes et al., 2020). Approximately 1.5 million cells from the in vitro infection culture were spun down at 400 x g for 5 min and resuspended in 45ul of PBS. Live/dead staining was performed using 5µl of a 1:60 dilution stock of prepared Live/Dead Fixable Aqua Dead Cell Stain (Invitrogen). Cells were stained for 5 minutes in the dark at room temperature. A staining cocktail with FACS buffer and CD8 BV570 (BioLegend #301038, clone RPA-T8) was added for a 10 minute stain in the dark at room temperature. 1ml FACS buffer was added and the cells were spun down at 400 x g for 5 min. Cells were permeabilized with 250µl of BD Cytofix/

	Cytoperm solution (BD #554714) for 18 minutes in the dark at room temperature. 1ml of BD Perm/Wash Buffer (BD #554714) was added and cells were spun down at 600 x g for 5 min. After supernatant was discarded, cells were resuspended in staining solution containing anti-p24 FITC (Beckman Coulter #6604665; clone KC57) and BD Perm/Wash Buffer for a final staining volume of 50µl. Cells were stained in the dark for 1 hour at room temperature. Cells were washed with 1ml of BD Perm/Wash Buffer and fixed with 350ul of 1% paraformaldehyde. 75345 events were acquired.
Instrument	BD FACS Symphony A5 cytometer
Software	FlowJo (v10.8.0)
Cell population abundance	Final population (p24+) was 5.10% (2546) of 49572 live singlet cells.
Gating strategy	Singlets (FSC-A by FSC-H) -> Live cells (negative staining for Live/Dead Aqua) -> p24+ CD8- T cells (p24+ and CD8-)

☒ Tick this box to confirm that a figure exemplifying the gating strategy is provided in the Supplementary Information.
